# Low-Dose Anti-HIV Drug Efavirenz Mitigates Retinal Vascular Lesions in a Mouse Model of Alzheimer’s Disease

**DOI:** 10.3389/fphar.2022.902254

**Published:** 2022-06-01

**Authors:** Nicole El-Darzi, Natalia Mast, David A. Buchner, Aicha Saadane, Brian Dailey, Georgios Trichonas, Irina A. Pikuleva

**Affiliations:** ^1^ Departments of Ophthalmology and Visual Sciences, Cleveland, OH, United States; ^2^ Departments of Genetics and Genome Sciences, Case Western Reserve University, Cleveland, OH, United States

**Keywords:** CYP46A1, retina, retinal angiomatous proliferation, efavirenz, retinal-choroidal anastomosis

## Abstract

A small dose of the anti-HIV drug efavirenz (EFV) was previously discovered to activate CYP46A1, a cholesterol-eliminating enzyme in the brain, and mitigate some of the manifestation of Alzheimer’s disease in 5XFAD mice. Herein, we investigated the retina of these animals, which were found to have genetically determined retinal vascular lesions associated with deposits within the retinal pigment epithelium and subretinal space. We established that EFV treatment activated CYP46A1 in the retina, enhanced retinal cholesterol turnover, and diminished the lesion frequency >5-fold. In addition, the treatment mitigated fluorescein leakage from the aberrant blood vessels, deposit size, activation of retinal macrophages/microglia, and focal accumulations of amyloid β plaques, unesterified cholesterol, and Oil Red O-positive lipids. Studies of retinal transcriptomics and proteomics identified biological processes enriched with differentially expressed genes and proteins. We discuss the mechanisms of the beneficial EFV effects on the retinal phenotype of 5XFAD mice. As EFV is an FDA-approved drug, and we already tested the safety of small-dose EFV in patients with Alzheimer’s disease, our data support further clinical investigation of this drug in subjects with retinal vascular lesions or neovascular age-related macular degeneration.

## Introduction

Alzheimer’s disease (AD), which affects the brain, is a major cause of dementia in humans (Winblad et al., 2016). Age-related macular degeneration (AMD), which affects the retina (Supplementary Figure S1A), is a major cause of legal blindness in the elderly of industrialized countries (Wong et al., 2014). AMD can manifest as a dry (non-neovascular) or wet (neovascular) form. The late stage of dry AMD is called geographic atrophy. Wet AMD is characterized by abnormal growth of blood vessels and is classified as type 1, 2, or 3 based on the origin and location of neovascularization (Supplementary Figure S1B). In type 1 AMD, the new blood vessels grow from the choroidal vascular network and remain below the layer of retinal pigment epithelial cells (RPE), which underlies the neural retina and rests on Bruch’s membrane (BM) (Freund et al., 2008; Freund et al., 2010). In type 2 AMD, the neovessels from the choroidal network break the BM/RPE complex and gain access to subretinal space, i.e., the interface between the RPE and photoreceptor outer segments (Freund et al., 2008; Freund et al., 2010). In type 3 AMD, also called retinal angiomatous proliferation (RAP), the blood vessels proliferate within and below the retina with recent studies suggesting that they originate from the retinal circulation (Li et al., 2018; Spaide, 2018; Breazzano et al., 2020). However, these neovessels do not always reach the choroid, hence the accuracy of the term “choroidal neovascularization (CNV)” is debatable for type 3 AMD (Spaide, 2018).

Notable hallmarks of AMD include extracellular lesions called drusen and subretinal drusenoid deposits (SDDs), which differ in location relative to the RPE, association with AMD types as well as lipid, protein, and mineral content (Curcio et al., 2005; Rudolf et al., 2008; Wang et al., 2010; Curcio, 2018a; Curcio, 2018b; Li et al., 2018; Spaide, 2018). In particular, drusen are found between the RPE basal lamina (basement membrane) and the inner collagenous layer of BM, whereas SDDs are located on the opposite side of the RPE - between the RPE and photoreceptors (Curcio, 2018a). Drusen are associated with geographic atrophy as well as types 1 and 2 neovascular AMD, while SDDs are recognized risk factors for types 2 and 3 AMD (Curcio, 2018b; Li et al., 2018; Spaide, 2018). Strikingly, SDDs are precursor lesions in 90% of eyes with type 3 neovascularization, the latter representing ∼34% of newly diagnosed cases of neovascular AMD (Chen et al., 2016). The major components of drusen are free (unesterified) and esterified cholesterol with lipid-containing particles occupying 37%–44% of the druse volume (Curcio et al., 2005; Wang et al., 2010). In contrast, the major lipid component in SDD is unesterified cholesterol (Oak et al., 2014). Drusen contain lipids and proteins common to extracellular deposits found in AD. Common lipids include cholesterol (Mori et al., 2001; Curcio et al., 2005; Wang et al., 2010; Oak et al., 2014) and common proteins include amyloid β peptides (Aβ) (Mullins et al., 2000; Crabb et al., 2002; Johnson et al., 2002; Luibl et al., 2006; Hoh Kam et al., 2010; Wang et al., 2010), a hallmark of AD that, like AMD, strongly correlates with advanced age and the formation of deposits.

At present, there are no disease-modifying treatments either for AD or AMD as all current therapies mainly target symptoms. This laboratory is focused on CYP46A1 (cytochrome P450 46A1) as a therapeutic target, a CNS-specific enzyme which converts cholesterol to 24-hydroxycholesterol (24HC) (Lund et al., 1999). In the brain, CYP46A1 controls the main pathway for cholesterol elimination and turnover (Lutjohann et al., 1996; Lund et al., 2003). In the retina, where CYP46A1 is expressed in some neurons of the ganglion cell layer and inner nuclear layer (INL) as well as the RPE (Bretillon et al., 2007; Ramirez et al., 2008; Zheng et al., 2012), this enzyme is similarly critical for keeping cholesterol levels normal (Saadane et al., 2019). In addition, CYP46A1 was shown to be important for retinal vascular permeability and repression of proinflammatory genes in retinal macrophages/microglia (Saadane et al., 2014; Saadane et al., 2019).

We discovered that a small dose of the FDA-approved anti-HIV drug efavirenz (EFV) allosterically activated CYP46A1 in mouse brain (Mast et al., 2014), and that EFV treatment of 5XFAD mice, a model of rapid amyloidogenesis in AD (Oakley et al., 2006), led to multiple brain effects. Not only was there an increase in brain cholesterol elimination and turnover in EFV-treated 5XFAD mice, but animal performance in memory tasks was improved as well. Additionally, there were treatment paradigm-specific changes in the brain Aβ load, expression of the brain synaptic proteins, brain inflammatory markers, brain transcriptome, proteome, and phosphoproteome (Mast et al., 2017; Petrov et al., 2019a; Petrov et al., 2019b; Mast et al., 2021). Herein, we describe the results of our retinal evaluations of 5XFAD, which we previously characterized for the brain EFV effects. Some of these 5XFAD mice appeared to develop retinal vascular lesions, whose incidence and appearance were improved after EFV treatment. The data obtained are of direct clinical relevance as our clinical study of EFV in patients with AD (ClinicalTrials.gov Identifier: NCT03706885) showed the safety of small-dose EFV and CYP46A1 engagement in human brain. Thus, EFV should be safe to be investigated in patients with neovascular AMD.

## Materials and Methods

### Animals and Treatments

Female and male mice were from the same cohorts of animals, which were characterized previously for the EFV brain effects (Mast et al., 2017; Petrov et al., 2019a). These were transgenic 5XFAD mice on the B6SJL background (5XFAD^*Tg/0*^, stock No: 34840**,** the Jackson Laboratory, Bar Harbor, ME, United States), hemizygous for the mutant (K670N, M671L, I716V, V717I) human amyloid precursor protein 695 and mutant (M146L and L286V) human presenilin 1 (Oakley et al., 2006). Males were crossed with wild type B6SJL females (stock No: 100012**,** the Jackson Laboratory) and only the F1 generation of hemizygous animals was then used. EFV was administered in drinking water containing 0.0004% Tween 80 at a 0.1 mg/kg body weight/day dose either from 1 to 9 months of age (1st treatment) or from 3 to 9 months of age (2nd treatment), i.e., a time point (9 months) when the Aβ production starts to plateau in this model (Oakley et al., 2006). Mice were euthanized during the last day of EFV administration. Accordingly, animals were 9 months of age at the end of experiments. Retinal *in vivo* imaging was carried out a day or two before the euthanasia, and the retina and brain were isolated immediately after the euthanasia followed by tissue processing. Control animals received aqueous 0.0004% Tween 80 (Mast et al., 2017; Petrov et al., 2019a). All animals were free of the *Crb1* ^*rd8*^ and *Pde6b* ^*rd1*^ mutations as confirmed by genotyping for these mutations as well as spectral-domain optical coherence tomography (SD-OCT) showing the lack of mutation-specific retinal lesions. *Crb1* ^*rd8*^ was never detected in our colony, and *Pde6b* ^*rd1*^ was bread out before animal crossing. Mice were maintained in a temperature and humidity-controlled environment with 12 h light/12 h dark cycle in cages with water and food *ad libitum*. All animal experiments were approved by the Case Western Reserve University’s Institutional Animal Care and Use Committee and conformed to recommendations by the American Veterinary Association Panel on Euthanasia.

### Retinal *In Vivo* Imaging

Retinal imaging by SD-OCT and fluorescein angiography (FA) was carried out as described (Omarova et al., 2012; Saadane et al., 2014). An Envisu R2200 UHR OCT imaging system (Leica Bioptigen, Morrisville, NC, United States) and a scanning laser ophthalmoscope (Spectralis HRA + OCT, Heidelberg Engineering, Franklin, MA, United States) were used, respectively, for each imaging modality. Images for FA were acquired after a bolus (0.1 ml) intraperitoneal injection of 1.0% sodium fluorescein (Akorn Inc., Lake Forest, IL, United States, #17478-250-20) in phosphate buffer saline (PBS).

### Retinal Sterol Measurements

These were as described (Mast et al., 2011; Saadane et al., 2014) using individual or pooled samples of retina plus RPE and isotope dilution gas chromatography-mass spectroscopy. The internal standards were deuterated sterol analogs. The quantifications were of total cholesterol (a sum of esterified and unesterified cholesterol) and of unesterified other sterols.

### Transmission Electron Microscopy

The posterior part of the eye was processed as described using the OTAP (osmium-tannic acid-para-phenylenediamine) technique (El-Darzi et al., 2021b). Briefly, the tissue was first fixed in a quarter strength of Karnovsky’s fixative (4% paraformaldehyde and 5% glutaraldehyde in 0.1 M Na cacodylate, pH 7.4) and then sequentially postfixed in 3% glutaraldehyde in 0.1 M Na cacodylate buffer, pH 7.4; 1% OsO_4_ in 0.1 M Na cacodylate buffer, pH 7.4; 1% tannic acid in 0.05 M Na cacodylate, pH 7.4; and 1% para-phenylenediamine in 70% ethanol. Thick (0.5 μM) sections were stained with toluidine blue (Electron Microscopy Sciences, Hatfield, PA, United States, #22050), and thin sections (70–80 nm) were stained with uranyl acetate (Electron Microscopy Sciences, #22400) and lead salts: acetate (Fisher Scientific, Waltham, MA, United States, #6080-56-4), nitrate (Mallinckrodt Chemicals, Phillipsburg, NJ, United States, #10099-74-8), and citrate (Fisher Scientific, #0-3372). Thin sections were examined by a 1200EX transmission electron microscope (JEOL Ltd., Japan).

### Histochemistry and Immunohistochemistry

The preparation of frozen sections and subsequent stains with filipin (Cayman Chemical, Ann Arbor, MI, United States, #70440), isolectin GS-IB4 conjugated to Alexa Fluor-594 (Invitrogen, Waltham, MA, United States, #121413), Oil Red O (StatLab, McKinney, TX, United States, #KTORO), Thioflavin S (MilliporeSigma, Burlington, MA, United States, #T1892) and rabbit polyclonal anti-Iba1 antibody (Wako, Richmond, VA, United States, #019-1974, 1:250 dilution) were as described (Omarova et al., 2012; Zheng et al., 2012; Saadane et al., 2014; Mast et al., 2017; El-Darzi et al., 2021a), except the incubation time for the Oil Red O binding was 17 min at 60°C. For vimentin staining, frozen retinal sections were blocked for 1 h at room temperature with 5% normal goat serum (Life Technologies, Frederick, MD, United States, PCN5000) and 0.05% Tween 20 (Fisher Scientific, Fair Lawn, NJ, United States, #BP337-500). Sections were then incubated overnight at 4°C with rabbit monoclonal anti-vimentin antibody (Abcam, Waltham, MA, United States, ab92547, 1:1,000 dilution) in PBS containing 5% normal goat serum and 0.05% Tween 20. The next morning, slides were washed three times for 5 min with PBS containing 0.05% Tween 20 and incubated for 1 h in the dark at room temperature with Alexa Fluor 647–conjugated goat anti-rabbit IgG (Jackson ImmunoResearch, West Grove, PA, United States; #111-605-144, 1:200 dilution) in blocking buffer. Sections were washed three times for 5 min with PBS, then dipped in distilled water, covered with DAPI Fluoromount-G (SouthernBiotech, Birmingham, AL, United States, #0100-20), and protected with a glass coverslip. For Aβ staining, brain and retinal frozen sections were warmed to room temperature and washed three times for 5 min with PBS. Brain sections were then subjected to antigen retrieval by 88% aqueous formic acid for 3 min at room temperature followed by a three-time (for 5 min) PBS wash; no antigen retrieval was carried out for retinal sections. Sections were then blocked for 1 h at room temperature with 5% normal goat serum and 0.3% Triton X-100 (Sigma Life Sciences, St. Louis, MO, United States, 9002-93-1) and incubated overnight at 4°C with the D54D2 XP rabbit monoclonal antibody Alexa Fluor 647 Conjugate (Cell Signaling Technology, Danvers, MA, United States #42284, 1:50 dilution) in PBS containing 1% bovine serum albumin (Sigma Life Sciences, St. Louis, MO, United States, A2153) and 0.3% Triton X-100. The next morning, slides were washed three times for 5 min with PBS, then dipped in distilled water, covered with DAPI Fluoromount-G, and protected with a glass coverslip.

### Retinal RNAseq

This was conducted by BGI Americas (Cambridge, MA, United States) as described (Jiang et al., 2019; Zhou et al., 2020) on three biological replicates per group, each representing a pooled sample of one retina plus RPE from five to six 9-month old 5XFAD male mice after the 2nd treatment (Petrov et al., 2019a). Briefly, total RNA was extracted by Trizol (Invitrogen, #15596026, Waltham, MA, United States) according to the manufacturer’s instructions. The RNA quality and quantity was assessed by a Nano Drop and Agilent 2100 bioanalyzer (Thermo Fisher Scientific, Waltham, MA, United States). The RNA was fragmented and reverse transcribed using random primers to obtain cDNA and construct the library. Library sequencing was carried out on the BGISEQ-300 platform with a sequencing depth of at least 30 million reads for each sample. The sequencing reads were filtered to obtain clean reads, which were stored in the FASTQ format. The Bowtie2 (v2.2.5) software was used to align clean reads to the mouse reference genome (UCSmm10). Gene expression was quantified by the Expectation Maximization (RSEM, v1.2.12) program and normalized to fragments per kilobase of exon model per million mapped reads (FPKM). The threshold for significant differential expression was based on the Poisson distribution with a fold change of ≥1.07 and a *p* value of ≤ 0.05.

### Retinal Proteomics

The label-free approach was used as described (Saadane et al., 2018) and was carried out by the Proteomics and Small Molecule Mass Spectrometry Core at Case Western Reserve University (Cleveland, OH, United States). Three biological replicates per genotype, each representing a pooled sample of one retina plus RPE from five to six 9-month-old 5XFAD male mice after the 2nd treatment (Petrov et al., 2019a) were used. Differences in relative protein abundance were calculated by the PEAKS software (Bioinformatics Solutions Inc. Waterloo, ON, Canada) based on unique peptides (Zhang et al., 2012). Proteins with non-significant changes (*p* > 0.05) in abundance between the groups were excluded from the subsequent analysis as were the proteins with less than 1.1-fold change in the relative abundance, even if this change was significant.

### Exome Sequencing and Variant Annotation

Exome sequencing was conducted by BGI Americas (Cambridge, MA, United States) as described (Banerjee et al., 2021) using genomic DNA prepared from liver samples using the QIAmp DNA Kit (#56304, Qiagen, Germany). An average of 87.1 million sequencing reads were obtained per sample (range: 56.6–103.2 million reads), and of them, an average of 92.9% of the sequencing reads had a quality score > Q30. The data for each sample were aligned to the mouse reference genome (mm10) using Burrows-Wheeler Aligner software (Li and Durbin, 2009; Li and Durbin, 2010) v0.7.15. All genomic variations, including SNPs and Indels (insertions and deletions) were detected by the HaplotypeCaller of the GATK (v3.6) software and using GATK best practices (Mckenna et al., 2010; Depristo et al., 2011). Then, the SnpEff tool was applied for variant annotation (Cingolani et al., 2012).

### Statistical Analyses

Data from all available retinas were used. There was no exclusion of statistical outliers. The sample size (n) and statistical analysis are indicated in each table/figure or table/figure legend. At least three animals per group were used. Only in the case of EFV-treated 5XFAD mice, studies by Transmission Electron Microscopy (TEM) and histo-/immunohistochemistry are representative of one randomly selected animal as a total of three animals with vascular lesions were found. Hence, we could allocate only one eye for each specific analysis to be able to characterize the retina by other methods. Data were analyzed either by Fisher’s exact two-tailed test or a two-tailed, unpaired Student’s *t*-test. The GraphPad Prism software (GraphPad) was used. Statistical significance was defined as ∗∗*p* ≤ 0.01 and ∗∗∗*p* ≤ 0.001.

## Results

### Retinal *In Vivo* Imaging

**FIGURE 1:**
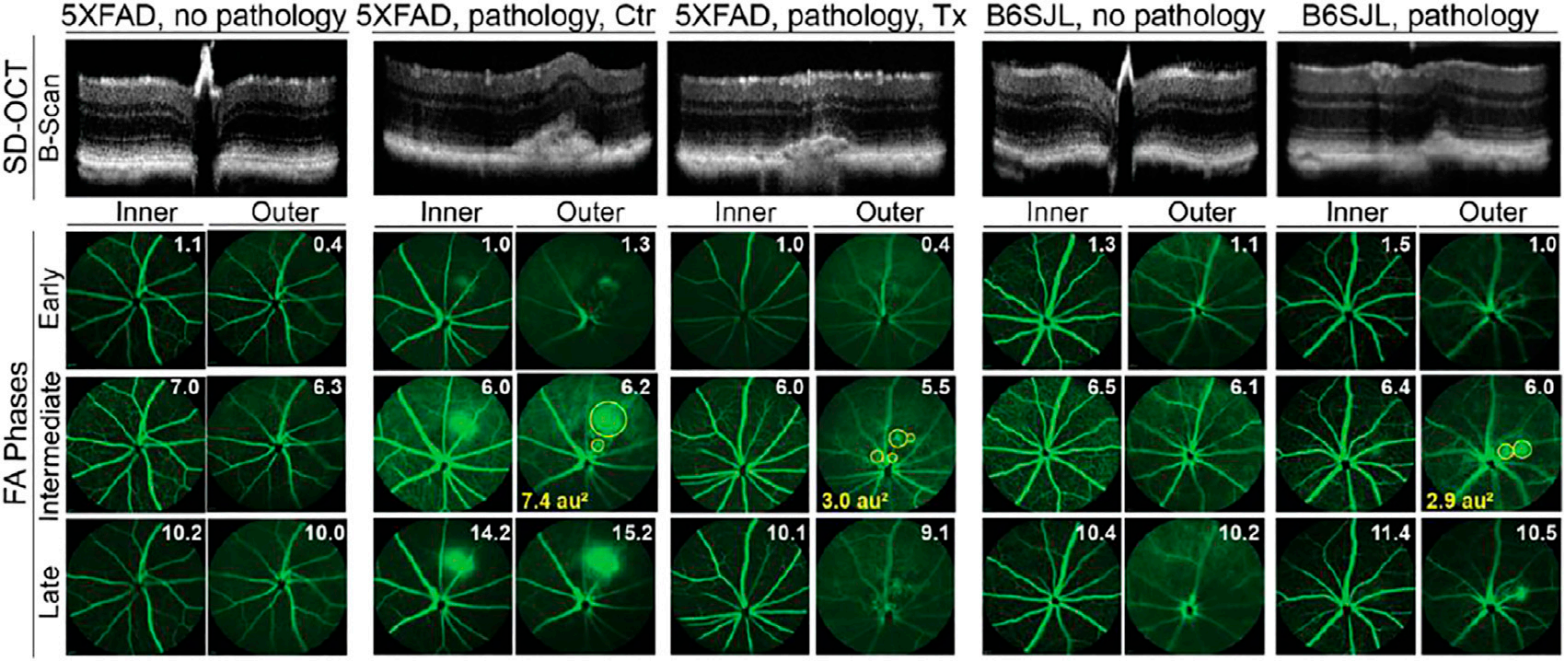
*In vivo* imaging of mouse retina. The spectral domain-optical coherence tomography (SD-OCT) panel shows retinal cross sections. Images are representative of 35 female and 27 male 5XFAD mice with no pathology [control (Ctr) and EFV-treated (Tx)]; eight female and six male control 5XFAD mice with pathology; 80 female and 104 male B6SJL mice with no pathology; and 10 female and 11 male B6SJL mice with pathology. B6SJL mice were used for the generation of hemizygous 5XFAD mice. The image of the EFV-treated mouse with pathology shows one (male mouse from the 1st treatment) of the two animals with the most pronounced EFV effect. The FA panel shows an early, intermediate, and late-stage fundus fluorescence (from top to bottom) as defined by the post-injection time of image acquisition (shown in minutes as white numbers in the upper right corner of each panel). Fluorescein leakage in the outer retina is outlined in the intermediate FA phase with yellow circles, and the circle area is calculated in arbitrary units (au) and shown in the lower left corner. The laser beam was focused on either the outer retina or inner retina, which are nourished by the retinal and choroidal vascular networks, respectively. The same animal was used for acquisition of SD-OCT and FA images. No sex-based differences were detected for pathology appearance on SD-OCT and FA.

Two cohorts of 5XFAD mice were evaluated (Table 1). Both received the same EFV dose (0.1 mg/day/kg of body weight) but had a different treatment duration: from 1 to 9 months of age in the 1st treatment and from 3 to 9 months of age in the 2nd treatment (Mast et al., 2017; Petrov et al., 2019a). SD-OCT revealed that 43% and 31% of control 5XFAD mice had a retinal structural abnormality (Figure 1) in the 1st and 2nd treatments, respectively (or 35% of mice in both treatments), mostly unilateral and mostly in one area superior to the optic nerve. This abnormality represented a focal elevation of the RPE and was filled with hyperreflective material.

**TABLE 1:** EFV treatment reduces the incidence of retinal lesions in 5XFAD mice.

Mouse inventory	1st treatment		2nd treatment		Total	
	Control	EFV-treated	Control	EFV-treated	Control	EFV-treated
Total number of mice	14 (14F^a^)	14 (10F + 4M^b^)	26 (9F + 17M)	34 (8F + 26M)	40 (23F + 17M)	48 (18F + 30M)
The number of affected mice	6 (6F)	1 (1M)	8 (2F + 6M)	2 (1F + 1M)	14 (8F + 6M)	3 (1F + 2M)
% of affected mice	43	7	31	6	35	6
*p* value^c^	0.08		0.01		0.0009	

There does not seem to be sex-based predilection of retinal pathology in 5XFAD mice as suggested by a similar % of pathology incidence in the groups of mice with very different female to male ratios (e.g., control or EFV-treated mice in the 1st and 2nd tretaments).

a Female mice.

b Male mice.

c As calculated by Fisher’s exact two-tailed test.

Next, we evaluated control 5XFAD mice by FA and imaging the retina at early, intermediate, and late FA phases (Figure 1). Fluorescein leakage was evident in this group in both early and late phases of the angiogram and was associated with the lesion on SD-OCT. The FA pattern was consistent with type 2 CNV in some mice or RAP leading to retinal-choroidal anastomosis (RCA) in other animals (Querques et al., 2012).

The assessment of EFV-treated 5XFAD mice (Figure 1), which were the littermates of the control mice, revelated that the incidence of retinal lesions in this group was much lower, 7% (*p* = 0.08) and 6% (*p* = 0.01) in the 1st and 2nd treatments, respectively, and 6% (*p* = 0.0009), if both treatments were combined. Notably, in the treated group, only three animals had retinal pathologies (Table 1). In two mice, the lesion size on SD-OCT and FA leakage were smaller as compared to the control group (Figure 1), and in one mouse, these pathologies were similar to those in untreated 5XFAD mice.

Previous studies of 5XFAD mice by others did not report retinal vascular lesions (Park et al., 2014; Parthasarathy et al., 2015; Park et al., 2017). Hence, we hypothesized that the detected pathology was genetically determined, i.e., was inherited from B6SJL female mice that were used for the generation of the hemizygous 5XFAD mice (see Materials and Methods). Indeed, screening by SD-OCT and then FA identified B6SJL mice, both males and females, with lesions looking similar to those in 5XFAD mice (Figure 1). Yet the lesion size was mostly smaller on SD-OCT and vascular leakage was not as prominent in the inner retina as in control 5XFAD mice.

### Sterol Profile in 5XFAD Mice

**FIGURE 2:**
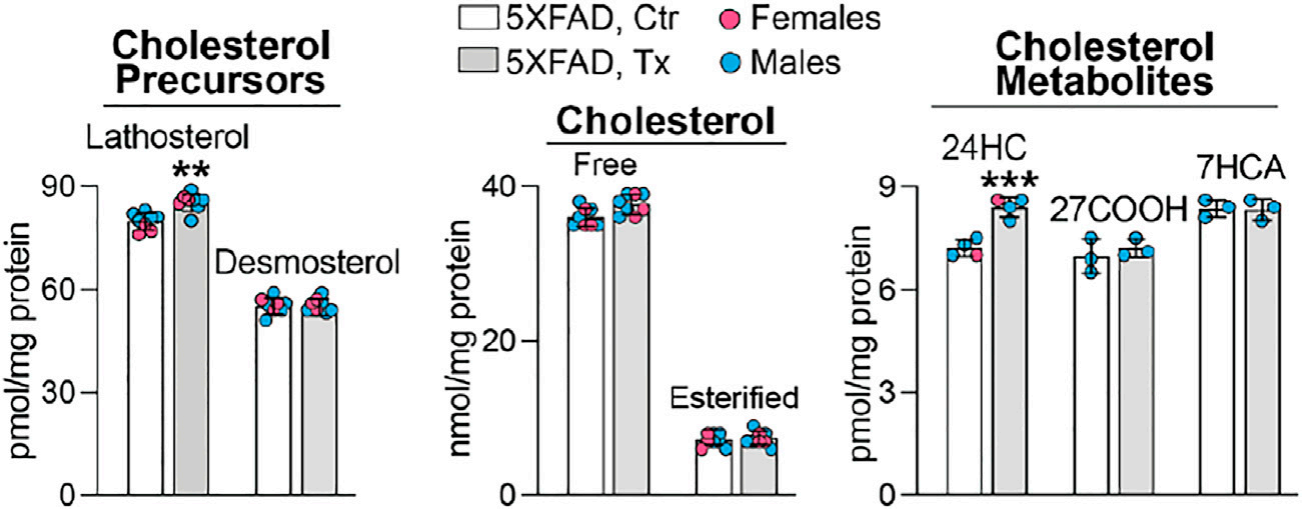
EFV effect on retinal sterol content in 5XFAD mice. Data represent the mean ±SD of the measurements either in individual retinas (lathosterol, desmosterol, and cholesterol: three females and five males) or in pooled retinas from 3 to 4 animals (24HC: one female and three male samples; and 27COOH and 7HCA: three male samples). No treatment or sex-based differences were detected. 24HC, 24-hydroxycholesterol; 27COOH, 5-cholestenoic acid; 7HCA, 7α-hydroxy-3-oxo-4-cholestenoic acid. Statistical significance was assessed by a two-tailed, unpaired Student’s *t*-test. **, *p* ≤ 0.01; ***, *p* ≤ 0.001.

To ascertain whether the beneficial EFV treatment effect could be linked to CYP46A1 activation and enhanced cholesterol turnover in the retina as it was in the brain of 5XFAD mice (Mast et al., 2017; Petrov et al., 2019a), six retinal sterols were quantified. These were cholesterol, two cholesterol precursors and markers of cholesterol biosynthesis in neurons (lathosterol) and astrocytes (desmosterol) (Nieweg et al., 2009; Mast et al., 2018), and three cholesterol metabolites, which are generated by CYP46A1 (24HC) and CYP27A1 [5-cholestenoic acid (27COOH) and 7α-hydroxy-3-oxo-4-cholestenoic acid (7HCA)], another cholesterol-metabolizing P450 in the retina (Lee et al., 2006; Omarova et al., 2012). Like in the brain of EFV-treated 5XFAD mice, retinal levels of total (free and esterified) cholesterol remained unchanged, whereas the 24HC and lathosterol levels were increased (Figure 2). This pattern of sterol changes was consistent with CYP46A1 activation in the retina and enhancement of retinal cholesterol turnover, as both retinal cholesterol biosynthesis and elimination were increased.

### Retinal Ultrastructure in the Lesion Area

**FIGURE 3:**
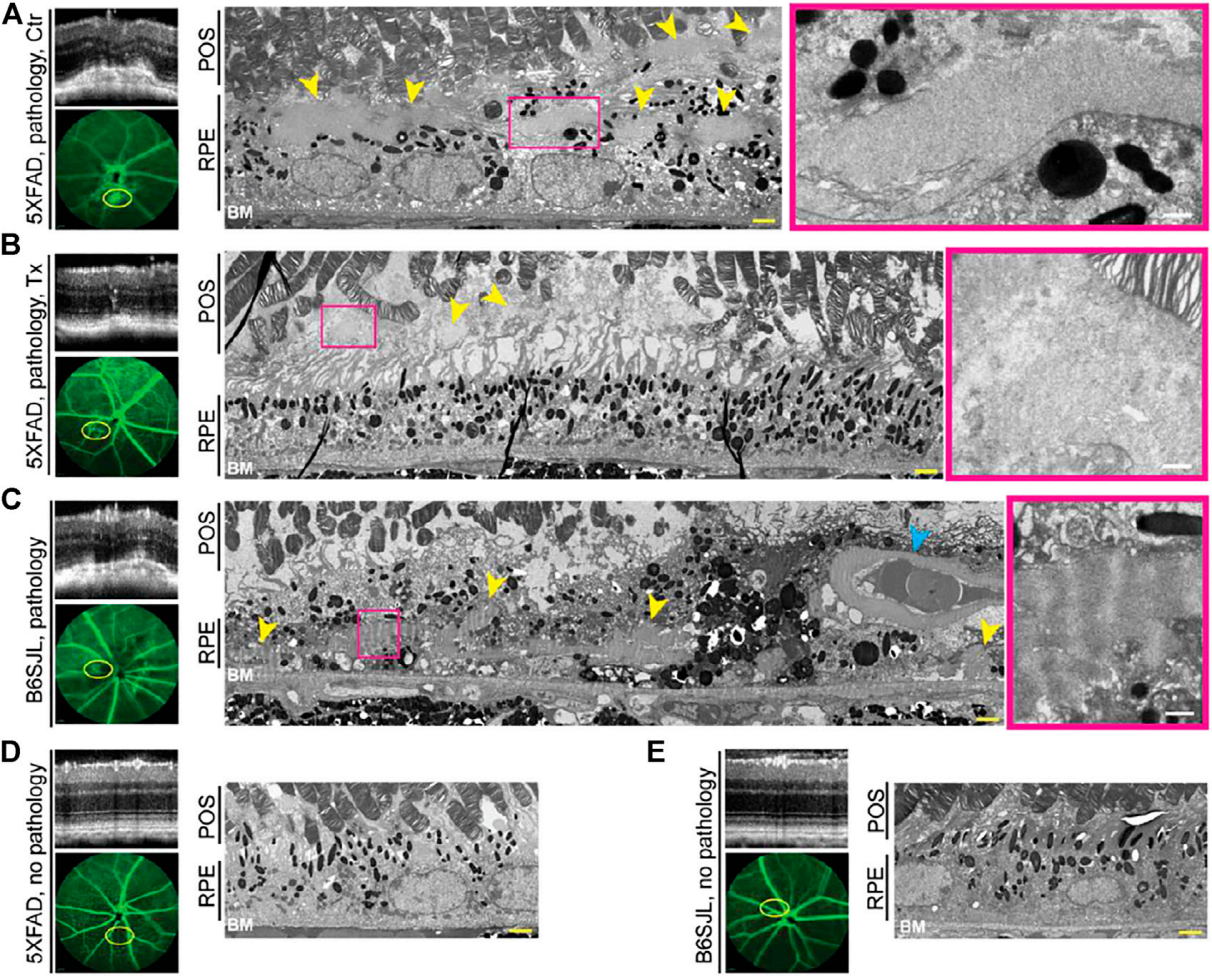
EFV effect on retinal deposits in 5XFAD mice **(A,B)**. Deposits in B6SJL mice **(C)** and retinal appearance in lesion-free 5XFAD **(D)** and B6SJL mice **(E)** are shown for comparison. Areas with or without pathology on SD-OCT (top left panels) were correlated to FA (intermediate phase, yellow ovals, left bottom panels) and tissue ultrastructure on TEM (two right panels). The same animal was used for acquisition of all the images in each panel. Images are representative of one randomly selected animal from each group. Yellow arrowheads denote deposits; cyan arrowhead denotes the abnormal blood vessel within the retinal pigment epithelium (RPE); and magenta rectangles outline a part of the retinal deposit that is shown at a higher magnification. POS, the photoreceptor outer segments; BM, Bruch’s membrane. Yellow scale bars are 2 μm; white scale bars are 0.5 μm.

Studies by TEM utilizing a lipid preservation technique (OTAP, see Materials and Methods) were carried out. In control 5XFAD retina, the area of vascular leakage had electron dense deposits both inside and outside the RPE (subretinally) (Figure 3A). However, in the EFV-treated 5XFAD retina, only deposit remnants were found, which confined subretinally (Figure 3B). These deposits were granulated and not as well defined or electron dense as in the control 5XFAD mouse. Deposits were also present in the lesion-containing B6SJL retina, mainly inside the RPE in proxy to the blood vessel, which normally should not be present in the RPE (Figure 3C). The RPE and BM below this blood vessel were intact, possibly because this blood vessel grew from the retinal circulation. Notably, the basement membrane of this abnormal blood vessel was very thick and seemed to be enriched with banded material resembling long spacing collagen as were adjacent deposits. A similar looking banded material is found in early basal laminar deposits in human aging RPE (Curcio and Millican, 1999; Sarks et al., 2007). Thus, neovascularization was correlated to deposits in the 5XFAD and B6SJL retina, and EFV treatment reduced the deposit size in the 5XFAD mouse. Yet, deposit location and appearance were not identical in the 5XFAD and B6SJL genotypes, possibly because the former was previously reported to accumulate intracellular Aβ in the RPE (Park et al., 2014; Park et al., 2017). Hence, we proceeded to investigate how Aβ plaques were distributed in the retina of different groups of mice.

### Amyloid β Peptides Plaques in Lesion-Free and Lesion-Containing Retinas

**FIGURE 4:**
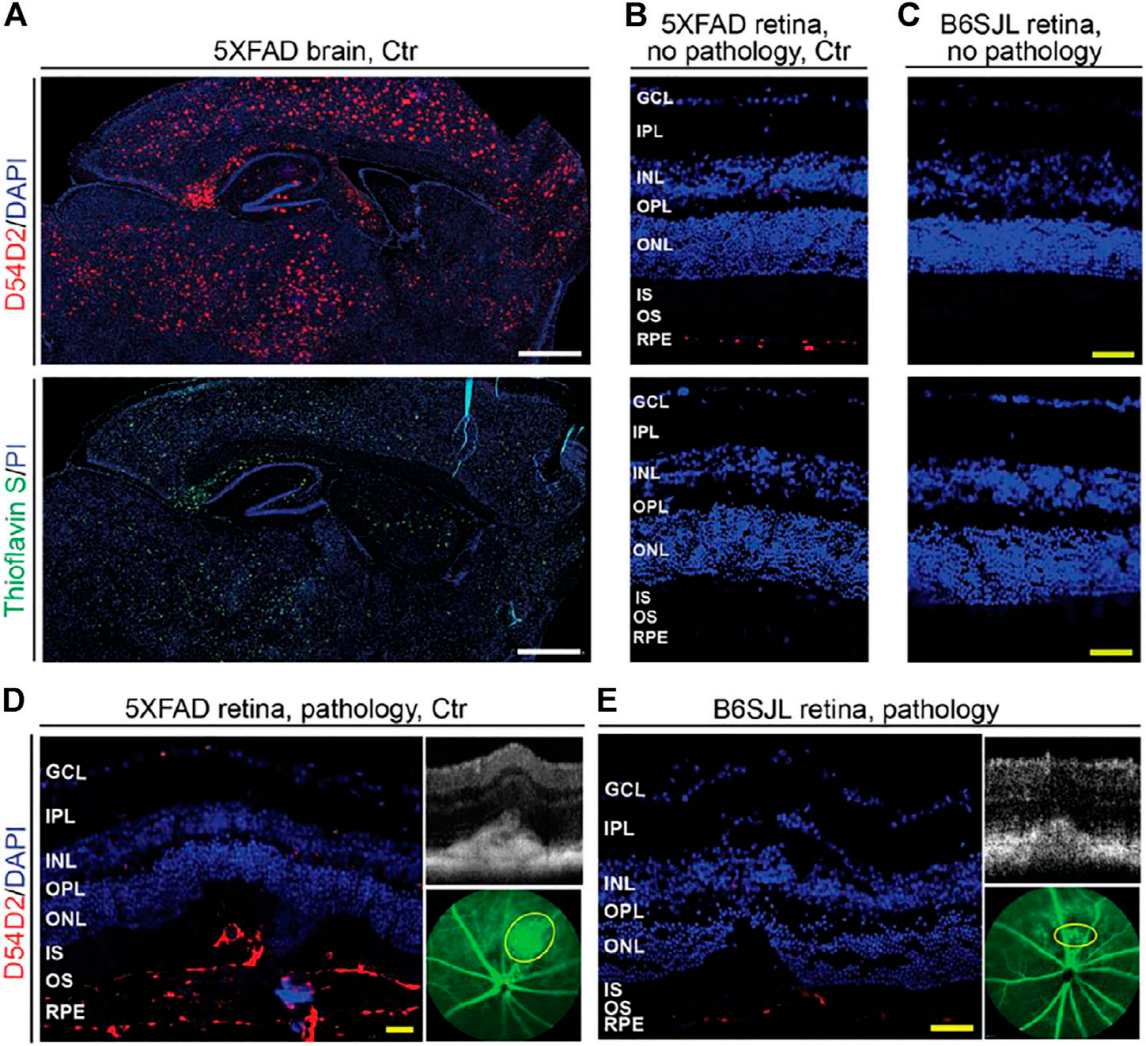
Aβ plaques in the brain **(A)** and retina **(B–E)** of 9-month old mice. Images are representative of 3 mice per group/panel. Nuclei were stained with DAPI or propidium iodide (PI); the latter was falsely colored in blue. **(D,E)** areas of pathology on SD-OCT (right top panels) were correlated to fluorescein angiography (intermediate phase, yellow ovals, right bottom panels) and then histology (left panels) for subsequent immunostaining with the D54D2 antibody; the same animal was used for acquisition of all the images. GCL, the ganglion cell layer; IPL, the inner plexiform layer; INL, the inner nuclear layer; OPL, the outer plexiform layer; ONL, the outer nuclear layer; IS, the photoreceptor inner segments; OS, the photoreceptor outer segments; RPE, the retinal pigment epithelium. White scale bars are 1,000 μm; yellow scale bars are 50 μm.

Stains with Thioflavin S and the D54D2 antibody were carried out. Thioflavin S binds to β sheet-rich structures and visualizes amyloid dense-core plaques observed in the later stages of AD (Dickson, 1997), whereas the D54D2 antibody interacts with Aβ peptides and detects amyloid diffuse-core plaques prevalent in the preclinical stages of the disease (Rozemuller et al., 1989). As compared to the 5XFAD brain, where both stains documented a significant Aβ load (Figure 4A), the abundance of Aβ plaques in the retina was much lower (Figures 4B,D,E). Aβ plaques were only detected with the D54D2 antibody and only in the 5XFAD retina (lesion-free and lesion-affected) as well as the lesion-affected B6SJL retina. There was no D54D2 immunoreactivity in the normal B6SJL retina (Figure 4C). Comparing the lesion-containing vs. lesion-free retinas, Aβ plaques were more abundant in the former in both 5XFAD and B6SJL retinas, where they were mainly found in the RPE and underlying choroid (Figures 4B,D) as well as in the subretinal space (Figures 4D,E). Thus, consistent with the 5XFAD genotype, retinal abundance of Aβ plaques was higher in 5XFAD mice than in B6SJL mice (with or without retinal lesions). Therefore, it is plausible that an increased Aβ load in the 5XFAD retina could modify the content of deposits in the lesion-containing regions and therefore exacerbate the frequency and manifestations of vascular lesions inherited from B6SJL mice. In addition, Aβ deposits could affect the integrity of the outer blood-retinal barrier as demonstrated previously in 5XFAD mice (Park et al., 2017), and thus facilitate the blood vessel growth through the BM/RPE complex.

### Lipid Deposition in the Lesion Area

**FIGURE 5:**
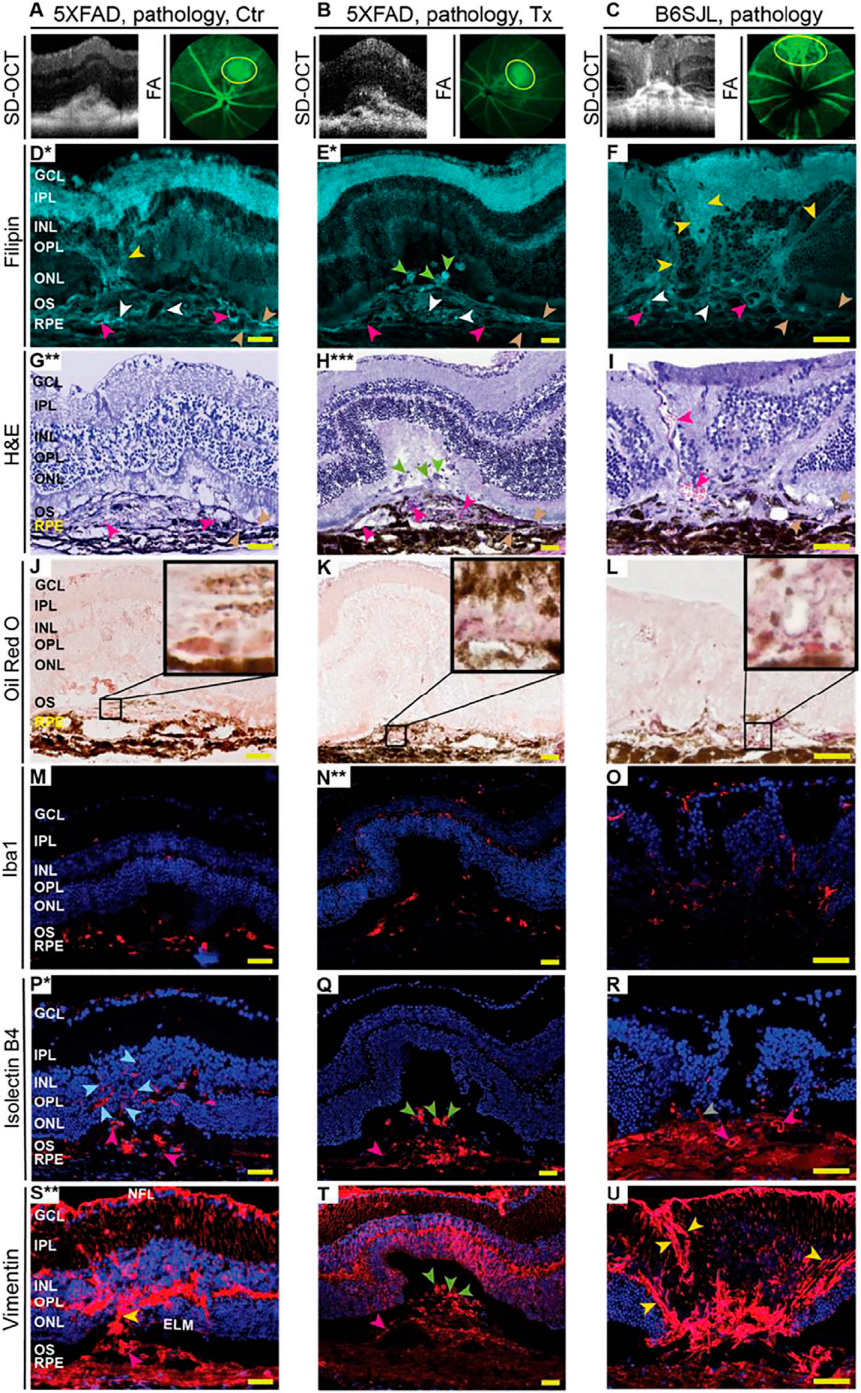
EFV effect on pathological processes in the retina in the lesion area. **(A**–**C)** Lesions on SD-OCT and fluorescein angiography (FA) were mapped on the retina, and the lesion area was then used for the generation of retinal cross sections and different types of stainings **(D**–**U)**. Serial sections from the same animal were used within each group of mice (each column). Due to the small size (0.05–0.1 mm), only a limited number of retinal serial sections (up to 10) could be cut through the lesion area. Therefore, only selected markers were assessed despite some sections being used two or even three times. Asterisks indicate sections (*) that were stained for the 2nd (**) or 3rd (***) time. Colored arrowheads denote some of the cell processes that were simultaneously filipin- and vimentin-positive (yellow); focal cholesterol deposits (white); blood vessels (magenta); the fragments of the apical and basal RPE membranes (wheat); filipin-, isolectin B4-, and vimentin-positive round structures in subretinal region (green); anastomosing blood vessels (light blue); and angiogenic sprout (grey). Images are representative of at least three mice per group/panel in the case of control 5XFAD mice and B6SJL mice and one animal in the case of the efavirenz-treated group. Nuclei were stained with DAPI or propidium iodide, and the latter was then falsely colored in blue. Scale bars are 50 μm.

To gain insights into the pathological processes in the retinal lesion area (Figures 5A–C), several markers were used, including those for lipids (filipin and Oil Red O), as lipids are significant components of drusen and subretinal drusenoid deposits (Curcio et al., 2005; Wang et al., 2010; Oak et al., 2014). Lipid histology was then correlated to retinal morphology either on the same or adjacent retinal sections stained with H&E.

Filipin binds to the 3β-hydroxyl group of unesterified cholesterol and labels all retinal layers but with different levels of intensity (Curcio et al., 2009; Pikuleva and Curcio, 2014). In the lesion area of control 5XFAD mice (Figure 5D), bright, focal filipin fluorescence was detected within the RPE disrupted by neovascularization, where the dye labeled fragments of the apical and basal RPE membranes, small cholesterol deposits, and the inner surface of some of the proliferating blood vessels. In addition, filipin stained cell processes that likely guided or served as a scaffold for the proliferating blood vessels from the deep capillary plexus (outer plexiform layer, OPL) toward the RPE. However, in the lesion area of EFV-treated 5XFAD mice (Figure 5E), the filipin labeling of the RPE basal and apical membrane fragments was less pronounced, focal cholesterol deposits were smaller in size, and the inner surface of the blood vessels within the RPE was not stained with filipin. Rather, there were several large and round filipin-positive structures in the subretinal space. In the lesion area of B6SJL mice (Figure 5F), the filipin staining pattern was similar to that of control 5XFAD mice as both focal cholesterol deposits, the inner surface of some of the blood vessels, and cell processes guiding the proliferating blood vessels were stained, although the latter also extended from the nerve fiber layer toward the INL. Filipin staining of these cell processes was not as clear as those originating from the OPL as it was confounded by the bright filipin fluorescence in the inner plexiform layer. The staining of the RPE plasma membranes was not intense as well as in the control 5XFAD retina.

Oil Red O binds to esterified cholesterol, triacylglycerides, free fatty acids, and retinyl esters (Malek et al., 2003; Oak et al., 2014), and, like filipin, stains all retinal layers but with different levels of intensity. Only very small regions (possibly lipid lakes in control 5XFAD retina and lipid droplets in B6SJL retina) were intensively labeled with Oil Red O in the lesion area in all animal groups (Figures 5J–L). The shape, size, and location of these intensively labeled regions varied within the group and between the groups, thus making comparisons difficult. The only notable difference was that EFV-treated 5XFAD mice were the only group in which Oil Red O-positive lipids formed clusters of small droplets (Figure 5K), and putative lipid lakes (control 5XFAD retina, Figure 5J) or large lipid droplets (B6SJL retina, Figure 5L), were not found. Thus, both filipin and Oil Red O stains identified focal lipid deposits in the lesion area in all groups of mice and suggested that these deposits are reduced after EFV treatment. In addition, filipin labeled round structures in the subretinal region of EFV-treated 5XFAD mice, which were absent in the other two groups of mice.

### Inflammation and Neovascularization in the Lesion Area

These processes were assessed by stainings for Iba1, vascular endothelial cells, and vimentin. Iba1 (ionized calcium-binding adaptor molecule 1) is specific for both resting and activated macrophages/microglia cells (Santos et al., 2008), simultaneously immune and angiogenic effector cells in the retina (Checchin et al., 2006; Rathnasamy et al., 2019). Indeed, upon activation, macrophages/microglia start to secrete proinflammatory cytokines and chemokines as well as pro-angiogenic factors, which elicit retinal inflammation and angiogenesis, respectively (Checchin et al., 2006; Rathnasamy et al., 2019). In control 5XFAD mice, 75% or 33 Iba1-positive cells had an amoeboid morphology, i.e., were activated, and were present in the region of the RPE disrupted by neovascularization (Figure 5M). Yet, in EFV-treated 5XFAD mice, only 24% or 18 Iba1-positive cells were activated and were mostly found below the photoreceptors along with the resting (ramified) Iba-positive cells (Figure 5N). Resting Iba1-positive cells were also present in the inner retina and OPL. Similar to EFV-treated mice, B6SJL mice had Iba-positive cells in almost every retinal layer (Figure 5O), and of them, 10% or 5 cells were activated (mostly present in the subretinal region). Thus, EFV-treatment seemed to reduce macrophage/microglia activation in 5XFAD mice, which could contribute in part to a reduction of neovascularization in this group.

Vascular endothelial cells were stained with isolectin B4, a carbohydrate-binding protein, which can also label some other cell types including activated microglia (Campos et al., 2006; Ernst and Christie, 2006; Tual-Chalot et al., 2013). Staining with isolectin B4 outlined the interior of some of the blood vessels in all three groups of mice and traced some of the retinal blood vessels, including the proliferating ones, in control 5XFAD mice as well as the angiogenic sprout in B6SJL mice (Figures 5P–R). Yet, we could not detect RAP in EFV-treated mice, rather we found that isolectin B4 labeled the round structures in the subretinal space in EFV-treated mice that were also stained with filipin (Figures 5E,Q). The shape of these structures did not resemble either endothelial or microglial cells and hence could represent the blood vessel remnants. We hypothesize that these structures could reflect reverse neovascularization or neovascularization regression as there were no isolectin B4-positive cells in the ONL of these mice, which were present in control 5XFAD and B6SJL mice. Thus, the isolectin B4 staining was consistent with RAP in some control 5XFAD and B6SJL mice and its absence in EFV-treated mice.

Vimentin (an intermediate filament protein) is a marker of mesenchymal-derived cells (e.g., fibroblasts and endothelial cells) as well as cells undergoing epithelial-mesenchymal or endothelial-mesenchymal transitions (Liu et al., 2015; Evrard et al., 2016; Piera-Velazquez and Jimenez, 2019), which play key roles in the pathogenesis of subretinal fibrosis, the end-stage of AMD (Shu et al., 2020). In the retina, vimentin is a cytoskeletal component in Muller and horizontal cells, astrocytes, and the walls of some blood vessels (Pérez-Álvarez et al., 2008). Staining for vimentin identified cell processes in all three groups of mice that were crossing the retina from the nerve fiber layer to the external limiting membrane and were likely from Muller cells (Figures 5S–U). Additionally, in control 5XFAD and B6SJL mice, there were cell processes that were both vimentin- and filipin-positive and that extended from the OPL towards the RPE. Plus in B6SJL mice, vimentin- and filipin-positive cell processers also extended from the nerve fiber layer toward the INL. Moreover, in one case, a vimentin- and filipin-positive cell process was clearly along the anastomosing blood vessel (Figures 5F,I,U).

Conversely, in EFV-treated 5XFAD mice, vimentin immunoreactivity in cell processes other than those from Muller cells was not apparent (Figure 5T). Rather, vimentin was detected in round structures in the subretinal space, some of which were stained with filipin and isolectin B, a finding which provides further support for our hypothesis about anastomosis regression and blood vessel remnants in subretinal space. As for the disrupted RPE region and underlying choroid, both had strong vimentin immunoreactivity in control 5XFAD and B6SJL mice but a weaker immunoreactivity in EFV-treated 5XFAD mice. This immunoreactivity could be due to the fibroblast staining and epithelial-mesenchymal or endothelial-mesenchymal transitions. Thus, the vimentin staining pattern was similar in control 5XFAD and B6SJL mice and different (except the Muller cells labeling) in EFV-treated mice. The data obtained suggested that vimentin is present in the cells involved in RAP, and that RCA could be absent in EFV-treated mice.

### Retinal Multiomics

To gain unbiased insights into the processes in the retina that could be affected by EFV treatment, two omics approaches were used (transcriptomics and proteomics) to compare the retinas of EFV-treated and control 5XFAD mice. Retinal RNAseq identified almost 19,000 genes expressed in both groups of mice (18,964 in control and 18,872 in EFV-treated group), and of them, 221 had a statistically significant change in the expression after EFV treatment. Of these 221 differentially expressed genes (DEGs), 120 were downregulated and 101 were upregulated in EFV-treated vs. control 5XFAD mice (Supplementary Table S1). The analysis of these DEGs by the PANTHER software (Mi et al., 2019) for statistical overrepresentation in the gene ontology (GO) biological processes identified 14 processes, in which either at least 10 DEGs were involved or the fold enrichment was no less than 5, both arbitrary cut offs (Table 2). The highest number of DEGs (24) was overrepresented in regulation of cell adhesion followed by regulation of proteolysis (20), apoptosis (14), blood vessel development (13), and other processes. The highest fold enrichment (11.3-8.9) was for energy metabolism (ADP metabolic, glycolytic, and pyruvate metabolic processes) as well as regulation of production of interleukin-10 (8.3). Remarkably, the latter is a cytokine that impairs the ability of ocular macrophages to regulate vascular endothelial cell proliferation and thus promotes choroidal neovascularization in mice (Apte et al., 2006).

**TABLE 2:** EFV effects on the retinal transcriptome of 5XFAD mice in the 2nd treatment.

DEGs/Total genes	Fold enrichment	GO biological processes and DEGs involved
**Regulation of cell adhesion (GO: 0030155)**		
24/773	3.2	*Agr2, Bst1, Cd47, Dock5, Dscam, Dusp1, Dusp26, Efnb2, Emilin1, Enpp2, Glmn, Gpnmb, H2-Ob, Hmgb1, Il4i1, Mbp, Mex3b, Mip, Nr4a3, Pkd1, Rag1, Sema3e, Slc7a1, Thbs1*
**Regulation of proteolysis (GO: 0030162)**		
20/740	2.8	*Alad, Clu, Eno1, Glmn, Hmgb1, Itih3, Mbp, Nkd2, Pcsk1n, Pi15, Pkd1, Prelid1, Prkaca, Rag1, Rnf144a, Styx, Tfap4, Thbs1, Trib2, Ubb*
**Blood vessel development (GO: 0001568) including regulation of angiogenesis GO: 0045765)**		
13/538	2.5	*Adgrf4, Col1a2, Col5a1, Col8a2, Edn2, Efnb2, Glmn, Jmjd6, Nat1, Pkd1, Sema3e, Stra6, Thbs1*
**Positive regulation of apoptotic process (GO: 0043065)**		
14/597	2.4	*Bmf, Clu, Dusp1, Egln1, Hmgb1, Nr4a3, Prelid1, Prkdc, Rpl26, Sik1, Tfap4, Thbs1, Ubb, Zmat3*
**Monocarboxylic acid metabolic process (GO: 0032787)**		
13/510	2.6	*Aldoa, Ech1, Edn2, Eno1, Fads,2 Il4i1, Lpin2, Nr4a3, Pfkfb2, Pfkl, Pkm, Rdh16f2, Tnxb*
**Visual system development (GO: 0150063)**		
12/393	3.2	*Ache, Bfsp2, Col5a1, Col8a2, Crybb1, Crybb3, Dscam, Hmgb1, Jmjd6, Mip, Rpe65, Stra6*
**Translation (GO:0006412)**		
11/323	3.5	*Mrps10, Rbm4, Rpl21, Rpl23a, Rpl26, Rpl28, Rpl35, Rpl41, Rplp0, Rps15, Rps28*
**Regulation of mitotic cell cycle (GO: 0007346)**		
11/456	2.5	*Bub1b, Cdc26, Gpnmb, Hmgb1, Kcnh5, Nup62, Pkd1, Prkdc, Rpl26, Sik1, Tfap4,*
**Negative regulation of immune system process (GO: 0002683)**		
11/460	2.5	*Cd47, Dusp1, Emilin1, Glmn, Gpnmb, H2-Ob, Hmgb1, Il4i1, Mmp28, Prkdc, Thbs1*
**ADP metabolic process (GO:0046031) including ATP generation from ADP (GO: 0006757)**		
6/55	11.3	*Aldoa, Ampd3, Eno1, Pfkfb2, Pfkl, Pkm*
**Glycolytic process (GO: 0009135)**		
6/58	10.7	*Aldoa, Ampd3, Eno1, Pfkfb2, Pfkl, Pkm*
**Pyruvate metabolic process (GO: 0006090)**		
6/70	8.9	*Aldoa, Eno1, Nr4a3, Pfkfb2, Pfkl, Pkm*
**Regulation of interleukin-10 production (GO: 0032653)**		
5/62	8.3	*Cd47, Hmgb1, Mbp, Trib2, Tusc2*
**Visual perception (GO:0007601) including sensory perception of light stimulus (GO: 0050953)**		
10/161	6.4	*Bfsp2, Crybb1, Crybb3, Guca1b, Lrat, Mip, Myo7a, Pde6g, Rpe65, Wfs1*

Statistical overrepresentation of the DEGs in the GO biological processes as identified by the PANTHER software (Mi et al., 2019).

Studies by proteomics identified a total of 2,644 proteins, of which 73 were differentially expressed (DEPs) in EFV-treated vs. control 5XFAD mice: 30 proteins were downregulated and 43 proteins were upregulated (Supplementary Table S2). Similar to transcriptomics, the highest number of DEPs (9) was overrepresented in the regulation of cell adhesion (Table 3). Yet there was no overlap between DEGs and DEPs in the latter as well as other biological processes, possibly because proteomics identifies only the most abundant retinal proteins. The other GO processes with a relatively high number of DEPs (no less than 5) included neuron development (8), regulation of vesicle-mediated transport (7), translation (6), and regulation of phagocytosis (6). The processes with the highest fold enrichment (no less than 7) included regulation of phagocytosis (15.2), RNA localization (8.8), regulation of proteasomal ubiquitin-dependent protein catabolic process (8.7), and protein dephosphorylation (7.5). Thus, the two multiomcs approaches identified meaningful processes in the retina that can be affected by EFV treatment.

**TABLE 3:** EFV effects on the retinal proteome of 5XFAD mice in the 2nd treatment.

DEPs/Total proteins	Fold enrichment	GO biological processes and DEPs involved
**Regulation of cell adhesion (GO: 0030155)**		
9/773	3.4	CD59A, COR2B, DUS3, EPHB2, FCL, MACF1, MYPT1, PTEN, TGM2
**Neuron development (GO: 0048666)**		
8/894	2.6	ATG7, EMB, EPHB2, GRK1, HCN1, NFH, **NRCAM,** PTEN
**Regulation of vesicle-mediated transport (GO: 0060627)**		
7/609	3.3	ATG7, LYAR, PLST, PTEN, RACK1, SNX3, TGM2
**Translation (GO: 0006412)**		
6/323	5.4	EIF3M, DENR, EIF3I, IF4G2, MCTS1, RACK1
**Regulation of phagocytosis (GO: 0050764)**		
6/114	15.2	ATG7, LYAR, PTEN, RACK1, SNX3, TGM2
**Regulation of cell development (GO: 0060284)**		
6/586	3.0	ATG7, EPHB2, MACF1, MGN, PTEN, TGM2
**Visual system development (GO: 0150063)**		
5/393	3.7	EPHB2, GRK1, HCN1, PDE6B, PDS5B
**RNA localization (GO: 0006403)**		
5/164	8.8	MGN, MGN2, NU214, RAE1L, SEC13
**Posttranscriptional regulation of gene expression including protein acetylation (GO: 0010608)**		
5/475	3.1	CNBP, IF4G2, MGN, RACK1, TADBP
**Regulation of microtubule-based process (GO: 0032886)**		
5/262	5.5	DCTN1, MACF1, MARE2, NFH, RAE1L
**Cell junction organization (GO: 0034330)**		
5/512	2.8	COR2B, DCTN1, EPHB2, PTEN, SC6A1
**Protein dephosphorylation (GO: 0006470)**		
4/238	7.5	DUS3, MYPT1, PP2AB, PTEN
**Regulation of epithelial cell migration (GO: 0010632)**		
4/238	4.9	FCL, MACF1, MARE2, PTEN
**Regulation of proteasomal ubiquitin-dependent protein catabolic process (GO: 0032434)**		
4/133	8.7	ATG7, RACK1, RD23B, UBP14
**Carboxylic acid catabolic process (GO: 0046395)**		
4/193	6.0	DDAH2, HMGCL, IVD, KBL
**Synapse organization (GO: 0050808)**		
4/306	3.8	DCTN1, EPHB2, PTEN, SC6A1

Statistical overrepresentation of the DEPs in the GO biological processes as identified by the PANTHER software (Mi et al., 2019).

### Exome Sequencing

**FIGURE 6:**
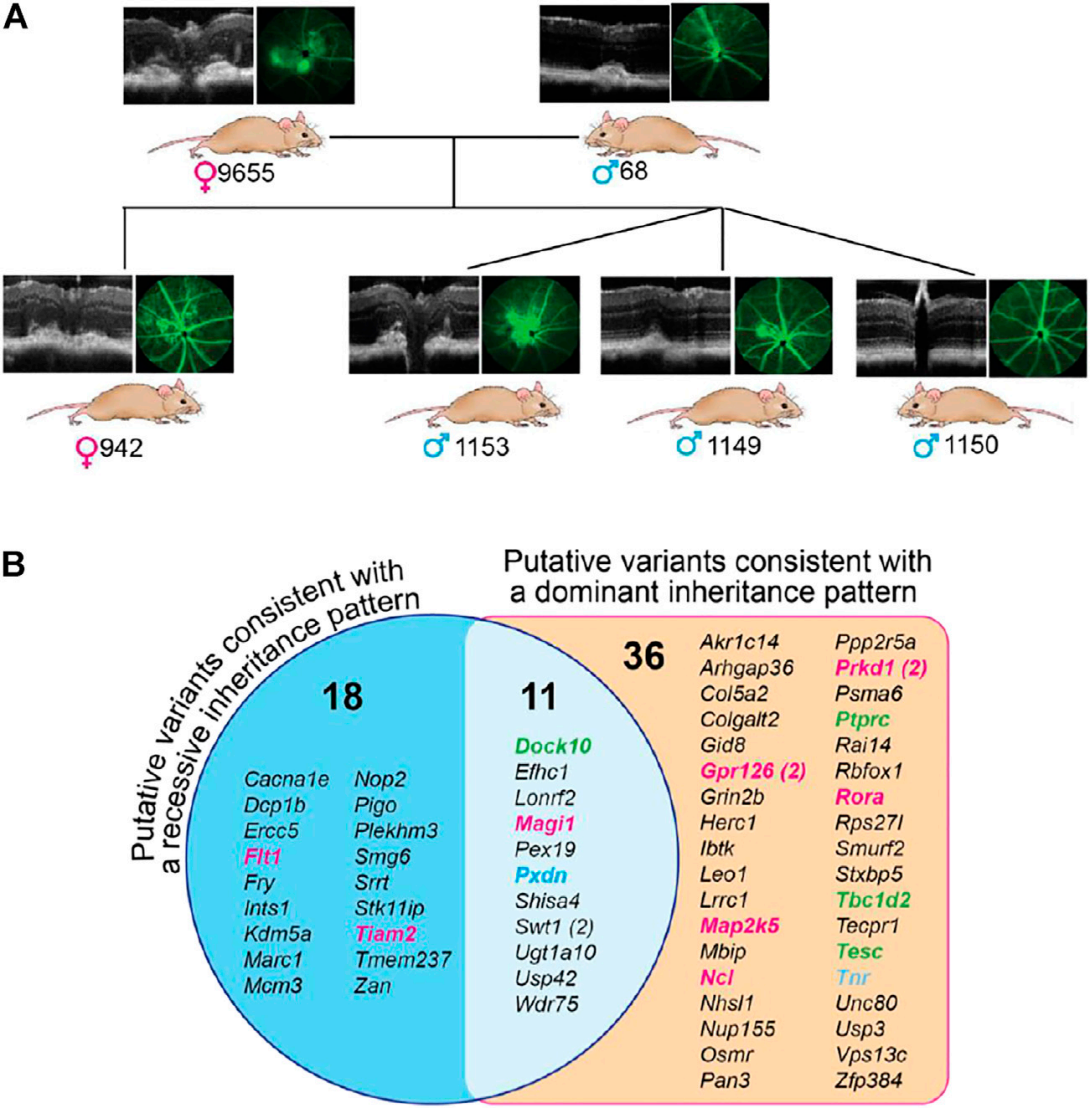
Exome sequencing of B6SJL mice with retinal lesions. **(A)**, Pedigree of mice used for exome sequencing. Mouse numbering is indicated on the right of the genetic sex symbol. **(B)**, Genes with common homozygous changes in mice with retinal lesions. The number in parenthesis indicates two different genetic variations. Genes that are colored are those involved in angiogenesis (magenta), cell-cell adhesion (green), and extracellular matrix formation (blue), the groups that can contribute to abnormal retinal phenotype of B6SJL mice. Several genes in these groups (from left to right columns) deserve particular consideration. Of the angiogenesis group, *Flt1* encodes the cell-surface receptor 1 for vascular endothelial growth factor (VEGF) **(A,B)**. Alternative splicing of *Flt1* yields soluble isoform, which acts as a trap for VEGFs and thereby blocks VEGF signaling, thus underlying corneal avascularity (Ambati et al., 2006; Singh et al., 2006; Ambati et al., 2007). Global *Flt1* deletion causes embryonic lethality in mice [64], whereas selective ablation of *Flt1* in the post-natal mouse retina leads to the hyperbranched vascular networks (Chappell et al., 2019). *Tiam2* encodes the Rac1 (a Rho GTPase)-specific guanine nucleotide exchange factor, a positive regulator of the vascular endothelium barrier function (Amado-Azevedo et al., 2017). *Magi1* encodes a membrane-associated guanylate kinase, also known as brain-specific angiogenesis inhibitor 1-associated protein. MAGI1 is a scaffold protein, which interacts with various proteins and thereby is involved in multiple cell functions including regulation of angiogenesis, vascular integrity, and permeability as well as cell–cell and cell–matrix adhesion (Wörthmüller and Rüegg, 2021). *Gpr126* encodes adhesion G-protein coupled receptor G6, which is highly enriched in vascular endothelial cells. GPR126 plays an important role in angiogenesis by regulating endothelial cell proliferation, migration, and tube formation (Cui et al., 2014). *Map2k5* encodes a dual specificity mitogen-activated protein kinase 5, a downstream target of VEGF. The signal cascade mediated by this kinase is involved in VEGF-induced cell proliferation, survival, and differentiation (Doebele et al., 2009; Huang et al., 2021). *Ncl* encodes cell surface nucleolin, which interacts with different ligands including VEGF-A and thereby regulates the endothelial cell activation and angiogenesis (Quiroz-Mercado et al., 2016; Darche et al., 2020). *Prkd1* encodes a protein kinase D1, which is involved in the regulation of multiple cellular processes including VEGFA-induced angiogenesis (Evans and Zachary, 2011; Steinberg, 2021). *Rora* encodes the retinoic acid receptor-related orphan receptor α, a lipid-sensing nuclear receptor with diverse biological functions including lipid metabolism, inflammation, and pathologic retinal angiogenesis (Sun et al., 2015). *Rora* is implicated in AMD pathogenesis (Silveira et al., 2010; Jun et al., 2011). Of the cell-cell adhesion group, *Dock10* (the dedicator of cytokinesis) encodes an exchange factor for the Rho GTPases RAC and CDC42 and, thereby, is involved in generic cell processes including the regulation of actin cytoskeleton, cell adhesion, and migration (Gadea and Blangy, 2014). Microglia migration is decreased in *Dock10* ^*−/−*^ mice (Namekata et al., 2020). *Ptprc* encodes protein tyrosine phosphatase receptor type C or CD45, which affects cell adhesion, migration, cytokine signaling, cell development, and activation state (Jonsson et al., 2021). *Tbc1d2* (or Armus) encodes a GTPase-activating protein for RAB7A GTPase. TBC1D2 acts as a linker between RAB7A and RAC1, leading to RAB7A inactivation and subsequent inhibition of cadherin degradation and reduced cell-cell adhesion (Frasa et al., 2010). Of the extracellular matrix formation group, *Pxdn* encodes a unique peroxidase, which stabilizes collagen IV networks and contributes to mechanical strength of basement membrane important for tissue integrity. PXDN mutations lead to severe eye disorders, including microphthalmia, cataract, glaucoma, and anterior segment dysgenesis in humans and mice (Yan et al., 2014; Kim et al., 2019). *Tnr* encodes tenascin-R, a neural extracellular matrix protein involved in interactions with different cells and matrix components that can influence cellular behavior by either evoking a stable adhesion and differentiation, or repulsion and inhibition of neurite growth (Pesheva et al., 1993; Pesheva et al., 1997; Probstmeier et al., 2000).

To gain insight into the genetic changes that could underlie retinal lesions in B6SJL mice, six animals were evaluated. Of them, five (two breeders and three F1 generation offspring) had retinal lesions, and one, the m1150 offspring, had normal retinas (Figure 6A). DNA isolated from the liver of these animals was subjected to exome sequencing, and the genes with homo- and heterozygous insertions, deletions, and single nucleotide polymorphisms were identified (Supplementary Tables S3, S4). These genetic changes were then analyzed for common presence in the investigated mice. In the first type of analysis, based on the assumption of a dominant inheritance pattern, we searched for common alleles in the five mice with retinal lesions, which had either hetero- or homozygous change for the same allele, and absent in the mouse with a normal retina. A total of 47 such genes were identified with variants that were predicted to alter the protein sequence of at least 1 transcript isoform. Some of the genes encoded proteins involved in angiogenesis (*Gpr126*, *Map2k5*, *Ncl*, *Prkd1*, and *Rora*), cell-cell adhesion (*Ptprc*, *Tbc1d2*, and *Tesc*), and extracellular matrix formation (*Tnr*) (Figure 6B). In the second type of analysis, based on the assumption of a recessive inheritance pattern, five mice with retinal lesions were examined for common alleles, which were all homozygous for an allele that was either heterozygous or not present in the mouse with normal retinas (Figure 6B). A total of 29 such genes were found with variants that were predicted to alter the protein sequence of at least 1 transcript isoform. Two of these genes encoded proteins involved in angiogenesis (*Flt1* and *Tiam2*). Notably, eleven variants were consistent with both recessive and dominant inheritance patterns as they were found in both analyses (Figure 6B). Of these variants, one was involved in angiogenesis (*Magi1*), one (*Dock10*) in cell-cell adhesion, and one (*Pxdn*) in extracellular matrix formation (Figure 6B).

**FIGURE 7:**
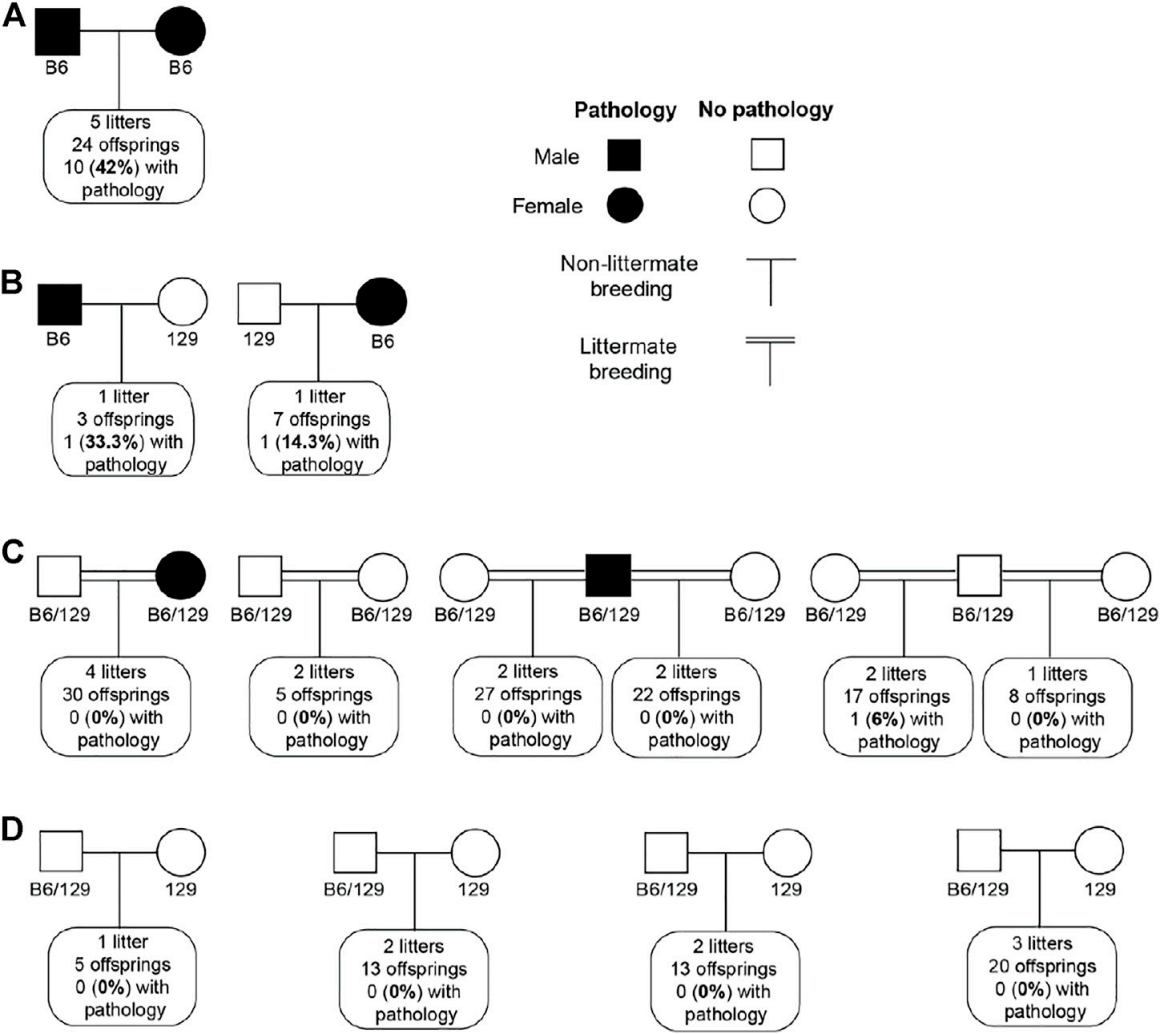
Inheritance pattern of retinal lesions in B6SJL (B6) mice. **(A)**, Cross between B6SJL mice exhibiting retinal pathologies. **(B)**, Offsprings of **(A)** with retinal pathologies backcrossed to 129S1/SvimJ wild type mice (129). **(C)**, Intercross of offsprings produced by **(B)**. **(D)**, Offsprings of **(C)** backcrossed to 129S1/SvimJ wild type mice.

We tried to establish the inheritance pattern of the retinal lesions in B6SJL mice. A cross between mice exhibiting the retinal pathology (the same mice used for exome analysis) produced 24 offspring from 5 l. Of them, 10%, or 42%, were mice that also had retinal lesions (Figure 7A). As this finding could be consistent with either a recessive or dominant inheritance pattern, additional crosses were performed. One male and one female offspring from these breeders, both of which had retinal lesions, were then backcrossed to 129S1/SvimJ wild type mice (Figure 7B). Among ten F1 offspring from this cross, 2 had retinal lesions (20%), potentially consistent with a dominant mode of inheritance. However, when these F1 offspring were intercrossed with each other, including the two offspring with retinal lesions, only 1 out of 109 (1%) of their F2 offspring exhibited retinal lesions. Notably, that one affected mouse was not from the cross with a parent with retinal pathology (Figure 7C). Furthermore, these F2 mice were then backcrossed with 129S1/SvimJ wild type mice, and no retinal lesions were detected among 51 offspring (Figure 7D). Collectively, these crosses suggested a complex polygenic inheritance pattern, with likely contributions from multiple B6 and/or SJL alleles.

## Discussion

In the present work, we conducted ophthalmic characterizations of 5XFAD mice that were previously investigated for EFV effects in the brain (Mast et al., 2017; Petrov et al., 2019a; Petrov et al., 2019b; Mast et al., 2020; Petrov et al., 2020; Mast et al., 2021). We established that, similar to the brain, EFV treatment increased CYP46A1 activity, cholesterol biosynthesis, and turnover in the retina (Figure 2) as well as affected retinal transcriptome and proteome (Tables 2, 3). Unexpectedly, we found that some of these animals developed subretinal and RPE deposits associated with neovascularization, and that EFV treatment mitigated both deposit size and vascular lesion frequency (Table 1) as well as vascular leakage on FA (Figures 1, 3, 5). In addition, there was a reduction in focal accumulations of Aβ plaques, unesterified cholesterol, and Oil Red O-positive lipids as well as changes in the shape and number of retinal macrophages/microglial cells found in the subretinal/RPE regions affected by neovascularization (Figure 5). Nevertheless, the composition of deposits and their major constituents still remain to be determined as do the cell types, whose processes guided the proliferating blood vessels in control 5XFAD and B6SJL mice. Some of these cell types could be horizontal cells as suggested by a study of a mouse model (*Vldlr* ^*−/−*^ mice) of RAP (Dorrell et al., 2009). This study demonstrated that the new blood vessel formation is associated with mistargeted neurites of horizontal cells, which serve as a template for vascular invasion into the ONL from the inner retina (Johnson et al., 2015). Also, it remains to be unambiguously determined the origin of filipin-, isolectin B4-, and vimentin-positive round structures in the subretinal region of EFV-treated 5XFAD mice (Figure 5) that we hypothesize could be remnants from regressing neovessels.

The present study demonstrates that in the brain and retina of the same mice, EFV exerted both organ- and pathology-specific effects as well as several similar effects—on CYP46A1 activity, cholesterol homeostasis, and macrophage/microglia activation. These similar effects could be due, at least in part, to an increased brain and retinal 24HC levels, a CYP46A1 enzymatic product and potent agonist of liver X receptors (LXRs) (Janowski et al., 1996; Chen et al., 2007). LXRs (LXRα and LXRβ) are transcription factors that serve as physiological regulators and integrators of cellular cholesterol uptake and removal, innate and adaptive immune responses, apoptosis, and phagocytosis (Zelcer and Tontonoz, 2006; Jakobsson et al., 2012). These effects are realized through LXR binding of 24HC or other oxysterols followed by either a transactivation or transrepression of target genes in a cell- and tissue-specific manner (Glass and Ogawa, 2006; Kalaany and Mangelsdorf, 2006; Shibata and Glass, 2010; Jakobsson et al., 2012; Spann and Glass, 2013). Genes that reduce cellular cholesterol load along with some of the genes important for phagocytosis (*Mertk*), apoptosis (*Aim, Lbp, and Mafb*) and reduction of inflammation (*Arg1*) are transactivated by LXRs (Kalaany and Mangelsdorf, 2006; Zelcer and Tontonoz, 2006; Calkin and Tontonoz, 2012; Lee and Tontonoz, 2015). In contrast, many pro-inflammatory genes (*Ccl2*, *Ccl4*, *Ccl7*, *Cox-2*, *Cxcl1*, *Il-1β, Il-6*, *iNos*, *Mmp9,* and *Tnfα*) are transrepressed by LXRs, mostly *via* NF-κB DNA-binding activity (Kalaany and Mangelsdorf, 2006; Zelcer and Tontonoz, 2006; Calkin and Tontonoz, 2012; Lee and Tontonoz, 2015).

Previously we established that LXRs are expressed in human retina and RPE (Zheng et al., 2012) and that many of the LXR transactivated genes are upregulated in the retina of mice treated with the synthetic LXR agonist TO901317 (Zheng et al., 2015). Conversely, we found that the LXR-transrepressed genes (*Ccl2*, *Cox-2*, *Cxcl1*, *Il-1β, iNos*, and *Tnfα*) are upregulated in the retina and retinal macrophages/microglial cells of animals lacking CYP46A1 (*Cyp46a1* ^*−/−*^ mice) (Saadane et al., 2019). Thus, we obtained evidence that LXRs regulate retinal gene transcription both *via* transactivation and transrepression and could be activated by 24HC, at least in retinal macrophages/microglial cells. In parallel, others demonstrated that in the retina or RPE, LXR expression is decreased in diabetic retinopathy and dry AMD and could contribute to lipid dysregulation in both diseases, possibly *via* LXRα in the latter (Hazra et al., 2012; Hammer et al., 2017; Choudhary et al., 2020). Studies on animal models and cell culture also suggested that both diseases could be mitigated, at least in part, by increased LXR activation with synthetic ligands (Hazra et al., 2012; Hammer et al., 2017; Choudhary et al., 2020).

In the present work, we did not find differential expression of the LXR target genes or their protein products either in the whole retinal transcriptome or proteome (Supplementary Tables S1, S2). This is possibly because these genes were affected only in a small subset of retinal cells (e.g., macrophages/microglia), and therefore changes in their expression could not be detected. Nevertheless, similar to treatments with synthetic LXR agonists (Hammer et al., 2017; Choudhary et al., 2020), we observed a reduction in retinal macrophage/microglia activation and lipid deposition within the RPE in EFV-treated 5XFAD mice (Figure 5). Obviously, further studies of control and EFV-treated 5XFAD mice that focus specifically on their retinal macrophages/microglia and LXRs target genes are required.

Can processes other than cholesterol removal and activation of LXRs or macrophages/microglia be affected by CYP46A1 activity in the retina? Probably, yes, as suggested by *CYP46A1*-containing adenoviral injections in the brain or EFV treatments of mouse models of different brain disorders, both neurodegenerative (e.g., Alzheimer’s, Huntington’s, and prion diseases, Niemann-Pick disease type C1, and spinocerebellar ataxia) and non-neurodegenerative (e.g., glioblastoma and depression) (Hudry et al., 2010; Burlot et al., 2015; Boussicault et al., 2016; Mast et al., 2017; Patel et al., 2017; Han et al., 2019; Kacher et al., 2019; Mitroi et al., 2019; Nobrega et al., 2019; Petrov et al., 2019a; Ali et al., 2021). In all these models, increases in CYP46A1 expression or activity were beneficial and targeted multiple processes and pathways (Pikuleva, 2021; Pikuleva and Cartier, 2021). Both common processes (e.g., abnormal protein accumulation, memory, motor functions, gene transcription, protein phosphorylation, autophagy, and lysosomal processing) and disease-specific processes were documented to be altered in the brain (Pikuleva, 2021; Pikuleva and Cartier, 2021). Hence, to integrate a broad range of CYP46A1 targeting outcomes in the brain, three CYP46A1-depenedent primary processes (called the unifying mechanisms) were proposed: sterol flux through the plasma membranes, acetyl-CoA production, and the mevalonate pathway (Supplementary Figure S2) (Petrov et al., 2020; Mast et al., 2021; Pikuleva, 2021; Pikuleva and Cartier, 2021).

Sterol flux was established to alter physico-chemical properties of plasma membranes and several membrane-dependent events (protein phosphorylation/dephosphorylation and synaptosomal glutamate release) (Petrov et al., 2020). Acetyl-CoA is known to be required for neurotransmitter biosynthesis, regulation of gene expression, cell death, mitosis, autophagy, cholesterol biosynthesis, and many other processes (Berg et al., 2002; Pietrocola et al., 2015). The mevalonate pathway [the first eight steps in cholesterol biosynthesis (Berg et al., 2002)] yields intermediates that are essential for protein prenylation and N-glycosylation, memory and motor functions, autophagy, lysosomal processing, cell survival, DNA replication, and mitochondrial electron transport (Pikuleva, 2021). Accordingly, like in the brain, EFV treatment could affect processes in the retina secondary to CYP46A1 activation as suggested by the changes in the retinal transcriptome and proteome in EFV-treated vs. control 5XFAD mice.

Indeed, several pathways under the three CYP46A1-depenedent primary processes were enriched with DEGs/DEPs in the retina (Tables 2, 3). These were glycolysis, pyruvate metabolism, ATP generation, and protein acetylation that determines acetyl-CoA levels along with mitosis, protein dephosphorylation, and non-LXR dependent apoptosis. In addition, there was DEGs/DEPs enrichment in the processes that directly related to angiogenesis - regulation of cell adhesion, blood vessel development, negative regulation of immune system process, regulation of interleukin-10 production, regulation of phagocytosis, and cell junction organization. Further studies are required to confirm retinal EFV effects on these processes.

EFV is not the first reverse transcriptase inhibitor among the anti-HIV drugs that was shown to exert a beneficial effect on a mouse model of AMD. Previously, intravitreal administrations of stavudine, lamivudine, zidovudine, and abacavir were demonstrated to suppress laser-induced choroidal neovascularization in mice (Fowler et al., 2014; Mizutani et al., 2015). Similarly, RPE degeneration (a hallmark of dry AMD) induced by subretinal injections to mice of Aβ was blocked by intravitreal co-administration of lamivudine and zidovudine (Narendran et al., 2021). Mechanistically, the tested reverse transcriptase inhibitors were discovered to possess intrinsic anti-inflammatory activity independent of their anti-viral function because they were found to inhibit P2RX7 (the purinergic P2X7 receptor), which is essential for activation of NLRP3 (the NLR family pyrin domain containing 3) inflammasome (Fowler et al., 2014; Mizutani et al., 2015; Narendran et al., 2021).

Herein, we did not test EFV for inhibition of P2RX7 and NLRP3 inflammasome. This was in part because we used a different EFV dose (0.1 mg/kg of body weight) and a different route of EFV administration (in drinking water). In studies of others, reverse transcriptase inhibitors were used at more than a 100 ng dose (Mizutani et al., 2015), which modelled a clinically relevant dose (>100 mg/day) in humans (Fowler et al., 2014), and the drug was injected intravitreally. Yet we do not exclude that the NLRP3 inflammasome activation could be suppressed in EFV-treated mice but *via* a different mechanism. This could be either *via* LXR activation or increased cholesterol removal by CYP46A1, as cellular cholesterol efflux was shown to mitigate the NLRP3 inflammasome activation induced by cholesterol accumulation (Lei et al., 2017; Yu et al., 2017; Westerterp et al., 2018; Zhang et al., 2021).

Recently, we concluded our clinical study of EFV in patients with AD (ClinicalTrials.gov Identifier: NCT03706885). Small doses of EFV were found to be safe for the geriatric participants and engaged CYP46A1 in the brain (the results will be published elsewhere). Accordingly, the present work is a pre-clinical study, whose results justify the investigation of EFV treatment in subjects with neovascular AMD. Moreover, EFV may even have some advantages over a potential use of the LXR synthetic agonists mechanistically. First, CYP46A1 activation by EFV not only increases the production of 24HC, a potent LXR endogenous ligand, but it also promotes the elimination of cellular cholesterol excess by metabolism, thus simultaneously affecting two processes. Second, CYP46A1 activation by EFV will only elicit the CNS-specific effects in humans, as in this species CYP46A1 is only expressed in the brain and retina (Lund et al., 1999). Conversely, a synthetic LXR agonist may have multiple systemic effects and lead to an undesired increase in serum triglyceride levels, the major current limitations of synthetic LXR agonists (Cao et al., 2004).

Finally, ophthalmic characterizations of 5XFAD mice by others showed intracellular Aβ accumulations in the RPE and suggested that these accumulations alter the RPE tight junctions, thus potentially contributing to the breakdown of the outer retinal blood-brain barrier (Park et al., 2014). Also, 5XFAD mice were shown to have ultrastructural changes in the RPE (an accumulation of lipofuscin granules and undigested photoreceptor outer segment-laden phagosomes along with a loss of apical microvilli and basal infolding) and BM (increased thickness, basal laminar and linear deposits) that are present in human dry AMD (Park et al., 2017). Changes in the retinal function were detected in 5XFAD mice as well (Lim et al., 2020). Accordingly, in the future, EFV evaluations could perhaps be expanded to the drug effects on these reported retinal changes.

In summary, we investigated the retina of 5XFAD mice, a model of rapid amyloidogenesis in AD, which developed subretinal and RPE deposits associated with retinal vascular lesions. 5XFAD mice treated with small-dose EFV, an anti-HIV drug, had increased activity of CYP46A1, a cholesterol-metabolizing enzyme in the retina, and enhanced retinal cholesterol turnover. The treated mice also had a reduction in deposit size and vascular lesion frequency along with the leakage on FA. The size of focal accumulations of Aβ plaques, unesterified cholesterol, and Oil Red O-positive lipids was also reduced as was the activation of retinal macrophages/microglial cells. We suggest that EFV treatment led to a regression of the neovascularization and that EFV treatment should be considered for further evaluations in patients with neovascular AMD.

## Data Availability

The datasets presented in this study can be found in online repositories. The names of the repository/repositories and accession number(s) can be found below: https://www.ncbi.nlm.nih.gov/geo; GSE199867; https://www.ncbi.nlm.nih.gov/sra; PRJNA821244.

## Abbreviations

24HC, 24-Hydroxycholesterol; Aβ, amyloid β peptides; AD, Alzheimer’s disease; AMD, age-related macular degeneration; BM, Bruch’s membrane; CNV, choroidal neovascularization; FA, fluorescein angiography; DEG, differentially expressed gene; DEP, differentially expressed protein; EFV, efavirenz; INL, inner nuclear layer; LXR, liver X receptor; OPL, outer plexiform layer; PBS, phosphate buffered saline; RAP, retinal angiomatous proliferation; RCA, retinal-choroidal anastomosis; RPR, retinal pigment epithelium; SDD, subretinal drusenoid deposits; SD-OCT, spectral-domain optical coherence tomography; TEM, transmission electron microscopy; VEGF, vascular endothelial growth factor.
