# Supplementary material for: Low-Dose Anti-HIV Drug Efavirenz Mitigates Retinal Vascular Lesions in a Mouse Model of Alzheimer’s Disease
Source: Front Pharmacol. 2022 Jun 1;13:902254. doi: 10.3389/fphar.2022.902254 (PMC9198296; doi:10.3389/fphar.2022.902254)
Supplement: Supplementary file 1 [file DataSheet1.PDF]

Supplementary Information

Low-dose anti-HIV drug efavirenz mitigates retinal vascular lesions in a mouse model of Alzheimer's disease

Nicole El-Darzi[1], Natalia Mast[1], David A. Buchner[2], Aicha Saadane[1], Brian Dailey[1], Georgios Trichonas[1], and Irina A. Pikuleva[1]*

Departments of [1]Ophthalmology and Visual Sciences and [2]Genetics and Genome Sciences, Case Western Reserve University, Cleveland, Ohio

Table S1. Differentially expressed genes ($P \leq 0.05$) in efavirenz-treated (Tx) retina *vs* control (Cntr) retina in 5XFAD mice as identified by RNAseq.

Gene	Ratio, Tx/Cntr	Gene	Ratio, Tx/Cntr	Gene	Ratio, Tx/Cntr
5730507C01Rik	1.24	*D130040H23Rik*	1.20	*Gm5415*	1.42
Abca13	1.17	*Ddost*	0.90	*Gng13*	0.90
Ache	1.09	*Dock5*	0.72	*Gng7*	1.10
Adgrf4	1.49	*Dpm3*	0.81	*Gpi1*	0.94
Agr2	0.70	*Drd4*	0.92	*Gpnmb*	1.45
Akap6	0.87	*Dscam*	0.90	*Gpr37l1*	1.56
Akr1e1	0.79	*Dusp1*	0.84	*Grem2*	1.29
Alad	0.86	*Dusp11*	1.07	*Gtf2a1*	1.11
Aldoa	0.92	*Dusp26*	1.09	*Guca1b*	1.10
Alpl	0.92	*Ech1*	1.28	*Gvin1*	1.59
Ampd3	0.84	*Edn2*	1.53	*H2-Ob*	1.76
Armh4	0.65	*Efnb2*	1.15	*Hmgb1*	0.91
Bcas1	1.77	*Egln1*	0.92	*Hsp90b1*	0.91
Bfsp2	0.64	*Egr2*	0.63	*Hspb2*	0.62
Bmf	0.80	*Emilin1*	1.42	*Igip*	1.19
Bspry	0.71	*Eno1*	0.90	*Igsf9*	1.16
Bst1	1.18	*Enox1*	0.71	*Il4i1*	0.60
Bub1b	0.89	*Enpp2*	1.14	*Insig1*	0.90
Calm2	0.93	*Entpd4b*	0.84	*Irf9*	1.59
Car12	1.48	*Esrrb*	0.84	*Itih3*	1.52
Cd47	1.12	*Etnppl*	1.41	*Jmjd6*	0.91
Cdc26	1.16	*Fads2*	0.89	*Kcnh5*	0.83
Cdin1	0.80	*Fam89b*	1.70	*Kcnj13*	1.45
Cldn23	1.24	*Fgd1*	1.13	*Kcnv2*	1.07
Clu	0.91	*Fkbp2*	0.84	*Kdm1b*	1.16
Cnrip1	0.91	*Frmpd1*	0.92	*Klhl28*	1.19
Col1a2	1.37	*Gdf15*	0.61	*Lbh*	0.90
Col5a1	1.21	*Gfap*	1.29	*Lgals6*	1.69
Col8a2	1.40	*Glmn*	1.14	*Lmbrd2*	1.14
Cox6b1	0.90	*Gm1140*	1.61	*Lmtk3*	1.10
Crybb1	0.68	*Gm11837*	0.55	*Lpin2*	0.89
Crybb3	0.66	*Gm12657*	1.49	*Lrat*	1.49
Cubn	0.66	*Gm14692*	0.55	*Lrrc8e*	0.72
Cyp4f16	0.77	*Gm45929*	0.72	*Mbp*	1.15

Mex3b	0.79	*Pon1*	1.47	*Slco5a1*	1.20
Mip	0.68	*Ppp1r3e*	1.36	*Smco3*	0.63
Mmp28	1.47	*Prelid1*	0.92	*Smim24*	0.92
Mobp	1.65	*Prkaca*	1.08	*Snhg11*	1.10
Mrps10	1.17	*Prkdc*	1.13	*Srp54a*	1.42
Mtrfr	0.84	*Psd*	1.08	*Srp54b*	1.26
Myl6	0.91	*Ptov1*	1.11	*Srp54c*	1.21
Myo15	1.52	*Rab6a*	0.93	*Sssca1*	0.85
Myo7a	0.80	*Rag1*	0.61	*Ssu72*	0.89
Nat1	0.74	*Rbm4*	0.80	*Stard7*	1.07
Ndufa13	0.91	*Rdh16f2*	0.70	*Stra6*	1.26
Nkd2	1.52	*Ric8b*	1.08	*Styx*	1.09
Nono	0.93	*Rmdn3*	0.89	*Supt16*	1.09
Nr4a3	0.69	*Rnf144a*	0.83	*Svep1*	1.69
Nudc	0.91	*Rpe65*	1.44	*Tead3*	1.15
Nudt3	0.92	*Rpl21*	0.82	*Tfap4*	0.77
Nup62	0.84	*Rpl23a*	0.93	*Thbs1*	1.33
Nutf2	0.86	*Rpl26*	0.78	*Tmem170*	1.27
Oasl2	1.24	*Rpl28*	0.89	*Tmem237*	0.91
Obscn	1.39	*Rpl35*	0.91	*Tmem40*	0.55
Odc1	1.13	*Rpl41*	0.87	*Tnxb*	1.28
Oog4	0.67	*Rpl7a*	0.92	*Tom1*	0.88
Opn3	0.76	*Rplp0*	0.91	*Trappc1*	0.87
Pabpc4	0.90	*Rps10*	0.89	*Trerf1*	1.17
Pcdha9	0.78	*Rps15*	0.93	*Trib2*	1.13
Pcdhga1	0.85	*Rps28*	0.87	*Trove2*	1.10
Pcsk1n	0.93	*Sap18b*	0.89	*Tspyl4*	0.93
Pde6g	0.91	*Sec61g*	0.84	*Tusc2*	0.91
Pfkfb2	1.07	*Sema3e*	0.87	*Tusc3*	1.12
Pfkfb3	0.88	*Sik1*	0.81	*Ubb*	0.91
Pfkl	0.90	*Sirt5*	0.81	*Ube2h*	1.09
Pgm2	0.92	*Six6os1*	1.16	*Ubl5*	0.92
Pi15	0.52	*Skp1a*	1.08	*Unc13c*	1.13
Pkd1	1.07	*Slc12a9*	1.14	*Wdr31*	0.88
Pkm	0.93	*Slc16a8*	1.40	*Wfs1*	0.92
Pla2g12a	1.36	*Slc29a1*	0.75	*Ythdf1*	1.13
Pnma3	0.79	*Slc30a3*	1.35	*Zan*	0.86
Pnp2	0.59	*Slc7a1*	0.81	*Zbtb20*	1.30

Zbtb43	1.13	*Zfp445*	1.07	*Zmat3*	1.18
Zfp383	1.15	*Zfp512b*	1.09		

Table S2. Differentially expressed proteins ($P \leq 0.05$) in efavirenz-treated (Tx) retina *vs* control (Cntr) retina in 5XFAD mice as identified by the label-free approach.

Protein name	# Unique peptides	Sequence Coverage (%)	Tx/Cntr ratio	Protein name	# Unique peptides	Sequence Coverage (%)	Tx/Cntr ratio
ATG7	2	6	0.33	MGN2	1	8	0.13
BZW1	1	7	0.14	MYPT1	2	3	0.67
CD59A	1	14	0.07	NCLN	3	7	0.43
CHKB	1	5	0.20	NDUB1	1	19	0.05
CNBP	3	22	0.14	NDUB7	4	36	0.11
COR2B	2	4	0.50	NFH	1	2	0.50
DCTN1	17	22	0.77	NMT1	5	25	0.20
DDAH2	9	59	0.15	NRCAM	10	13	0.77
DENR	2	12	0.17	NT5C	3	26	0.12
DHX30	1	1	1.00	NU214	1	1	1.00
DUS3	3	24	0.13	PAQR4	1	5	0.20
EIF3I	3	15	0.20	PDE6B	20	47	0.43
EIF3M	2	8	0.25	PDS5B	1	1	1.00
ELMD2	2	9	0.22	PGRC2	4	41	0.10
EMB	2	7	0.29	PLST	5	12	0.42
EMC8	2	13	0.15	PP2AB	1	43	0.02
EPHB2	7	10	0.70	PPIB	5	26	0.19
FCL	3	15	0.20	PTEN	1	4	0.25
GMEB1	1	5	0.20	RAB6A	1	12	0.08
GMPR1	1	4	0.25	RACK1	13	57	0.23
GRK1	21	49	0.43	RAE1L	2	8	0.25
GSHR	6	22	0.27	RD23B	2	7	0.29
GSTT2	1	5	0.20	RN170	1	5	0.20
HCN1	4	8	0.50	SAFB1	4	14	0.29
HMGCL	3	15	0.20	SC6A1	6	15	0.40
IF4G2	5	12	0.42	SCPDL	3	16	0.19
IVD	2	6	0.33	SEC13	5	23	0.22
KBL	3	13	0.23	SFXN5	6	29	0.21
LY6H	1	9	0.11	SNX3	3	25	0.12
LYAR	5	18	0.28	SP16H	4	6	0.67
MACF1	11	3	3.67	SPCS1	1	7	0.14
MARE2	7	39	0.18	SRSF5	1	9	0.11
MCTS1	3	28	0.11	TADBP	8	33	0.24

MGN	1	8	0.13	TGM2	1	2	0.50
THYN1	4	18	0.22	VAT1	4	15	0.27
TRIM2	3	6	0.50	WBP2	1	4	0.25
UBP14	5	20	0.25				

Supplemental Table S3. List of variants consistent with a dominant inheritance pattern.

<table>
<tr><th></th><th></th><th></th><th></th><th></th><th></th><th></th><th colspan="6">Genotype</th><th></th><th></th><th></th><th></th></tr>
<tr><th>Chr</th><th>Start (bp)</th><th>End (bp)</th><th>Gene</th><th>Ref allele</th><th>Alt allele</th><th>dbSNP</th><th>1153</th><th>68</th><th>942</th><th>1149</th><th>9655</th><th>1150</th><th>Annotation (primary transcript)</th><th>Annotation (alt transcript)</th><th>Transcript: Variant</th><th>Transcript isoforms with annotation</th></tr>
<tr><td>chr1</td><td>20990590</td><td>20990590</td><td>Efhc1</td><td>C</td><td>CTT</td><td>rs260911513</td><td>hom</td><td>hom</td><td>hom</td><td>hom</td><td>hom</td><td></td><td>intron_variant</td><td>protein_coding</td><td>NM_027974:c.1876-26_1876-25insTT</td><td>intron_variant(MODIFIER|||c.1876-26_1876-25insTT|648|Efhc1|protein_coding|CODING|NM_027974|10|1)</td></tr>
<tr><td>chr1</td><td>38812397</td><td>38812397</td><td>Lonrf2</td><td>AAC</td><td>A</td><td>rs226794629</td><td>hom</td><td>hom</td><td>hom</td><td>hom</td><td>hom</td><td></td><td>intron_variant</td><td>protein_coding</td><td>NM_001029878:c.653+145_653+146delGT</td><td>intron_variant(MODIFIER|||c.653+145_653+146delGT|518|Lonrf2|protein_coding|CODING|NM_001029878|5|1)</td></tr>
<tr><td>chr1</td><td>45391638</td><td>45391638</td><td>Col5a2</td><td>GA</td><td>G</td><td>rs387070045</td><td>het</td><td>hom</td><td>het</td><td>het</td><td>het</td><td></td><td>intron_variant</td><td>protein_coding</td><td>NM_007737:c.2709+23delT</td><td>intron_variant(MODIFIER|||c.2709+23delT|1497|Col5a2|protein_coding|CODING|NM_007737|40|1)</td></tr>
<tr><td>chr1</td><td>45805341</td><td>45805341</td><td>Wdr75</td><td>G</td><td>GTT</td><td></td><td>hom</td><td>hom</td><td>hom</td><td>hom</td><td>hom</td><td></td><td>intron_variant</td><td>protein_coding</td><td>NM_028599:c.496-35_496-34insTT</td><td>intron_variant(MODIFIER|||c.496-35_496-34insTT|830|Wdr75|protein_coding|CODING|NM_028599|5|1)</td></tr>
<tr><td>chr1</td><td>66626400</td><td>66626400</td><td>Unc80</td><td>C</td><td>CTT</td><td></td><td>hom</td><td>het</td><td>het</td><td>hom</td><td>het</td><td></td><td>intron_variant</td><td>protein_coding</td><td>NM_175510:c.5353-27_5353-26insTT</td><td>intron_variant(MODIFIER|||c.5353-27_5353-26insTT|3326|Unc80|protein_coding|CODING|NM_175510|33|1)</td></tr>
<tr><td>chr1</td><td>80578770</td><td>80578770</td><td>Dock10</td><td>TACACACAC</td><td>T</td><td></td><td>hom</td><td>hom</td><td>hom</td><td>hom</td><td>hom</td><td></td><td>intron_variant</td><td>protein_coding</td><td>NM_175291:c.1686-70_1686-63delGTGTGTGT</td><td>intron_variant(MODIFIER|||c.1686-70_1686-63delGTGTGTGT|2187|Dock10|protein_coding|CODING|NM_175291|14|1)</td></tr>
<tr><td>chr1</td><td>86351577</td><td>86351577</td><td>Ncl</td><td>TTA</td><td>T</td><td>rs387876869</td><td>het</td><td>hom</td><td>hom</td><td>het</td><td>hom</td><td></td><td>intron_variant</td><td>protein_coding</td><td>NM_010880:c.1697-86_1697-85delTA</td><td>upstream_gene_variant(MODIFIER||293|||Snora75||NON_CODING|NR_028478||1),intron_variant(MODIFIER|||c.1697-86_1697-85delTA|707|Ncl|protein_coding|CODING|NM_010880|11|1)</td></tr>
<tr><td>chr1</td><td>88056440</td><td>88056440</td><td>Ugt1a10</td><td>G</td><td>GTTTTT</td><td></td><td>hom</td><td>hom</td><td>hom</td><td>hom</td><td>hom</td><td></td><td>intron_variant</td><td>protein_coding</td><td>NM_201641:c.855+105_855+106insTTTTT</td><td>intron_variant(MODIFIER|||c.855+105_855+106insTTTTT|530|Ugt1a10|protein_coding|CODING|NM_201641|1|1)</td></tr>
<tr><td>chr1</td><td>135371901</td><td>135371901</td><td>Shisa4</td><td>A</td><td>AAAAAC</td><td>rs246361051</td><td>hom</td><td>hom</td><td>hom</td><td>hom</td><td>hom</td><td></td><td>3_prime_UTR_variant</td><td>protein_coding</td><td>NM_175259:c.*48_*49insGTTTT</td><td>3_prime_UTR_variant(MODIFIER||48|c.*48_*49insGTTTT|197|Shisa4|protein_coding|CODING|NM_175259|5|1)</td></tr>
<tr><td>chr1</td><td>138113783</td><td>138113783</td><td>Ptprc</td><td>G</td><td>GA</td><td></td><td>het</td><td>het</td><td>het</td><td>het</td><td>het</td><td></td><td>splice_region_variant</td><td>protein_coding</td><td>NM_001111316:c.611_612insT</td><td>splice_region_variant(LOW|||c.611_612insT|1293|Ptprc|protein_coding|CODING|NM_001111316|7|1),splice_region_variant(LOW|||c.122_123insT|1130|Ptprc|protein_coding|CODING|NM_001268286|4|1),splice_region_variant(LOW|||c.194_195insT|1154|Ptprc|protein_coding|CODING|NM_011210|5|1),intron_variant(MODIFIER|||c.611-4_611-5insT|1293|Ptprc|protein_coding|CODING|NM_001111316|7|1),intron_variant(MODIFIER|||c.122-4_122-5insT|1130|Ptprc|protein_coding|CODING|NM_001268286|4|1),intron_variant(MODIFIER|||c.194-4_194-5insT|1154|Ptprc|protein_coding|CODING|NM_011210|5|1)</td></tr>
<tr><td>chr1</td><td>151368380</td><td>151368380</td><td>Swt1</td><td>T</td><td>TTTTTTTTTC</td><td>rs216922920</td><td>hom</td><td>hom</td><td>hom</td><td>hom</td><td>hom</td><td></td><td>3_prime_UTR_variant</td><td>protein_coding</td><td>NM_025819:c.*78_*79insGAAAAAAAA</td><td>3_prime_UTR_variant(MODIFIER||78|c.*78_*79insGAAAAAAAA|907|Swt1|protein_coding|CODING|NM_025819|20|1)</td></tr>
<tr><td>chr1</td><td>152495887</td><td>152495887</td><td>Colgalt2</td><td>GTC</td><td>G</td><td></td><td>hom</td><td>het</td><td>hom</td><td>hom</td><td>hom</td><td></td><td>intron_variant</td><td>protein_coding</td><td>NM_177756:c.1026+10_1026+11delTC</td><td>intron_variant(MODIFIER|||c.1026+10_1026+11delTC|625|Colgalt2|protein_coding|CODING|NM_177756|7|1)</td></tr>
<tr><td>chr1</td><td>159884682</td><td>159884682</td><td>Tnr</td><td>A</td><td>AAG</td><td></td><td>het</td><td>het</td><td>het</td><td>het</td><td>het</td><td></td><td>intron_variant</td><td>protein_coding</td><td>NM_022312:c.1964-88_1964-87insAG</td><td>intron_variant(MODIFIER|||c.1964-88_1964-87insAG|1358|Tnr|protein_coding|CODING|NM_022312|9|1)</td></tr>
<tr><td>chr1</td><td>172133959</td><td>172133959</td><td>Pex19</td><td>C</td><td>CTTT</td><td></td><td>hom</td><td>hom</td><td>hom</td><td>hom</td><td>hom</td><td></td><td>intron_variant</td><td>protein_coding</td><td>NM_023041:c.772-40_772-39insTTT</td><td>intron_variant(MODIFIER|||c.496-40_496-39insTTT|207|Pex19|protein_coding|CODING|NM_001159525|4|1),intron_variant(MODIFIER|||c.772-40_772-39insTTT|299|Pex19|protein_coding|CODING|NM_023041|6|1)</td></tr>
<tr><td>chr1</td><td>191352925</td><td>191352925</td><td>Ppp2r5a</td><td>C</td><td>CA</td><td>rs258134793</td><td>het</td><td>het</td><td>het</td><td>het</td><td>het</td><td></td><td>3_prime_UTR_variant</td><td>protein_coding</td><td>NM_144880:c.*51_*52insT</td><td>3_prime_UTR_variant(MODIFIER||51|c.*51_*52insT|486|Ppp2r5a|protein_coding|CODING|NM_144880|13|1),downstream_gene_variant(MODIFIER||1||350|Tmem206|protein_coding|CODING|NM_025864||1)</td></tr>
<tr><td>chr2</td><td>180717794</td><td>180717794</td><td>Gid8</td><td>TG</td><td>T</td><td></td><td>het</td><td>het</td><td>het</td><td>het</td><td>het</td><td></td><td>intron_variant</td><td>protein_coding</td><td>NM_029607:c.514-43delG</td><td>intron_variant(MODIFIER|||c.514-43delG|228|Gid8|protein_coding|CODING|NM_029607|3|1)</td></tr>
<tr><td>chr4</td><td>46629779</td><td>46629779</td><td>Tbc1d2</td><td>ACACACACAC</td><td>T</td><td>rs252890567</td><td>hom</td><td>het</td><td>het</td><td>hom</td><td>hom</td><td></td><td>intron_variant</td><td>protein_coding</td><td>NM_198664:c.790+81_790+96delTGTGTGTGTGTGTGTG</td><td>intron_variant(MODIFIER|||c.790+81_790+96delTGTGTGTGTGTGTGTG|922|Tbc1d2|protein_coding|CODING|NM_198664|4|1)</td></tr>
<tr><td>chr5</td><td>118046248</td><td>118046248</td><td>Tesc</td><td>ATCTCTC</td><td>A</td><td>rs258321763</td><td>het</td><td>hom</td><td>hom</td><td>hom</td><td>het</td><td></td><td>intron_variant</td><td>protein_coding</td><td>NM_021344:c.59-66_59-61delTCTCTC</td><td>intron_variant(MODIFIER|||c.59-66_59-61delTCTCTC|214|Tesc|protein_coding|CODING|NM_021344|1|1)</td></tr>
<tr><td>chr5</td><td>143721062</td><td>143721062</td><td>Usp42</td><td>TAA</td><td>T</td><td>rs249277515</td><td>hom</td><td>hom</td><td>hom</td><td>hom</td><td>hom</td><td></td><td>intron_variant</td><td>protein_coding</td><td>NM_029749:c.875+84_875+85delTT</td><td>intron_variant(MODIFIER|||c.875+84_875+85delTT|1324|Usp42|protein_coding|CODING|NM_029749|8|1)</td></tr>
<tr><td>chr5</td><td>144218469</td><td>144218469</td><td>Tecpr1</td><td>T</td><td>TACAC</td><td></td><td>hom</td><td>hom</td><td>hom</td><td>het</td><td>hom</td><td></td><td>intron_variant</td><td>protein_coding</td><td>NM_027410:c.225+45_225+44insGTGT</td><td>intron_variant(MODIFIER|||c.225+45_225+44insGTGT|1166|Tecpr1|protein_coding|CODING|NM_027410|2|1)</td></tr>
<tr><td>chr5</td><td>147526800</td><td>147526800</td><td>Pan3</td><td>CT</td><td>C</td><td>rs261040637</td><td>hom</td><td>het</td><td>hom</td><td>hom</td><td>hom</td><td></td><td>intron_variant</td><td>protein_coding</td><td>NM_028291:c.1411+121delT</td><td>intron_variant(MODIFIER|||c.1411+121delT|837|Pan3|protein_coding|CODING|NM_028291|9|1)</td></tr>
<tr><td>chr6</td><td>93701094</td><td>93701094</td><td>Magi1</td><td>AAAC</td><td>A</td><td></td><td>hom</td><td>hom</td><td>hom</td><td>hom</td><td>hom</td><td></td><td>intron_variant</td><td>protein_coding</td><td>NM_001029850:c.2568+193_2568+195delGTT</td><td>intron_variant(MODIFIER|||c.2568+193_2568+195delGTT|1471|Magi1|protein_coding|CODING|NM_001029850|16|1),intron_variant(MODIFIER|||c.1884+193_1884+195delGTT|1255|Magi1|protein_coding|CODING|NM_001083320|15|1),intron_variant(MODIFIER|||c.1887+193_1887+195delGTT|1020|Magi1|protein_coding|CODING|NM_001083321|15|1),intron_variant(MODIFIER|||c.2445+193_2445+195delGTT|1171|Magi1|protein_coding|CODING|NM_010367|14|1)</td></tr>
<tr><td>chr6</td><td>125010499</td><td>125010499</td><td>Zfp384</td><td>CT</td><td>C</td><td>rs225093781</td><td>hom</td><td>hom</td><td>hom</td><td>het</td><td>hom</td><td></td><td>intron_variant</td><td>protein_coding</td><td>NM_175557:c.-6+60delT</td><td>intron_variant(MODIFIER|||c.-6+60delT|554|Zfp384|protein_coding|CODING|NM_001252083|2|1),intron_variant(MODIFIER|||c.-6+60delT|584|Zfp384|protein_coding|CODING|NM_175557|2|1)</td></tr>
<tr><td>chr6</td><td>135732070</td><td>135732070</td><td>Grin2b</td><td>ATC</td><td>A</td><td></td><td>het</td><td>het</td><td>hom</td><td>het</td><td>hom</td><td></td><td>3_prime_UTR_variant</td><td>protein_coding</td><td>NM_008171:c.*26_*27delGA</td><td>3_prime_UTR_variant(MODIFIER||26|c.*26_*27delGA|1482|Grin2b|protein_coding|CODING|NM_008171|15|1)</td></tr>
<tr><td>chr9</td><td>63358105</td><td>63358105</td><td>Map2k5</td><td>C</td><td>CT</td><td>rs263420860</td><td>hom</td><td>het</td><td>het</td><td>het</td><td>het</td><td></td><td>intron_variant</td><td>protein_coding</td><td>NM_011840:c.185-45_185-46insA</td><td>intron_variant(MODIFIER|||c.185-45_185-46insA|448|Map2k5|protein_coding|CODING|NM_011840|2|1)</td></tr>
<tr><td>chr9</td><td>66463170</td><td>66463170</td><td>Herc1</td><td>GCT</td><td>G</td><td></td><td>hom</td><td>het</td><td>het</td><td>het</td><td>het</td><td></td><td>intron_variant</td><td>protein_coding</td><td>NM_145617:c.9164+74_9164+75delCT</td><td>intron_variant(MODIFIER|||c.9164+74_9164+75delCT|4859|Herc1|protein_coding|CODING|NM_145617|45|1)</td></tr>
<tr><td>chr9</td><td>66547959</td><td>66547959</td><td>Usp3</td><td>C</td><td>CAAAAAAAA</td><td></td><td>hom</td><td>het</td><td>het</td><td>het</td><td>het</td><td></td><td>splice_region_variant</td><td>protein_coding</td><td>NM_144937:c.285_286insTTTTTTTT</td><td>splice_region_variant(LOW|||c.285_286insTTTTTTTT|520|Usp3|protein_coding|CODING|NM_144937|3|1),intron_variant(MODIFIER|||c.285-4_285-5insTTTTTTTT|520|Usp3|protein_coding|CODING|NM_144937|3|1)</td></tr>
<tr><td>chr9</td><td>66947704</td><td>66947704</td><td>Rps27l</td><td>TTTTTTTTTT</td><td>C</td><td></td><td>hom</td><td>het</td><td>het</td><td>het</td><td>het</td><td></td><td>splice_region_variant</td><td>protein_coding</td><td>NM_026467:c.227_239delTTTTTTTTTTTTT</td><td>splice_region_variant(LOW|||c.227_239delTTTTTTTTTTTTT|84|Rps27l|protein_coding|CODING|NM_026467|3|1),intron_variant(MODIFIER|||c.226+8_226+20delTTTTTTTTTTTTT|84|Rps27l|protein_coding|CODING|NM_026467|3|1)</td></tr>
<tr><td>chr9</td><td>67899903</td><td>67899903</td><td>Vps13c</td><td>TAA</td><td>T</td><td></td><td>hom</td><td>het</td><td>het</td><td>het</td><td>het</td><td></td><td>intron_variant</td><td>protein_coding</td><td>NM_177184:c.2405+1154_2405+1155delAA</td><td>intron_variant(MODIFIER|||c.2405+1154_2405+1155delAA|3748|Vps13c|protein_coding|CODING|NM_177184|24|1)</td></tr>
<tr><td>chr9</td><td>68769386</td><td>68769386</td><td>Rora</td><td>G</td><td>GT</td><td></td><td>het</td><td>het</td><td>het</td><td>het</td><td>het</td><td></td><td>intron_variant</td><td>protein_coding</td><td>NM_013646:c.166+115415_166+115416insT</td><td>intron_variant(MODIFIER|||c.166+115415_166+115416insT|523|Rora|protein_coding|CODING|NM_013646|1|1),non_coding_exon_variant(MODIFIER|||n.4042_4043insT||9530091C08Rik||NON_CODING|NR_033299|1|1)</td></tr>
<tr><td>chr9</td><td>75450595</td><td>75450595</td><td>Leo1</td><td>CTT</td><td>C</td><td>rs232490069</td><td>hom</td><td>het</td><td>het</td><td>het</td><td>het</td><td></td><td>intron_variant</td><td>protein_coding</td><td>NM_001039522:c.1248+24_1248+25delTT</td><td>intron_variant(MODIFIER|||c.1248+24_1248+25delTT|667|Leo1|protein_coding|CODING|NM_001039522|6|1)</td></tr>
<tr><td>chr9</td><td>77452779</td><td>77452779</td><td>Lrrc1</td><td>G</td><td>GT</td><td></td><td>hom</td><td>het</td><td>het</td><td>het</td><td>het</td><td></td><td>intron_variant</td><td>protein_coding</td><td>NM_001146048:c.788-58_788-59insA</td><td>intron_variant(MODIFIER|||c.788-58_788-59insA|524|Lrrc1|protein_coding|CODING|NM_001146048|8|1),intron_variant(MODIFIER|||c.653-58_653-59insA|479|Lrrc1|protein_coding|CODING|NM_172528|8|1)</td></tr>
<tr><td>chr9</td><td>85737385</td><td>85737385</td><td>Ibtk</td><td>TAA</td><td>T</td><td>rs245690359</td><td>hom</td><td>het</td><td>het</td><td>het</td><td>het</td><td></td><td>intron_variant</td><td>protein_coding</td><td>NM_001081282:c.418+81_418+82delTT</td><td>intron_variant(MODIFIER|||c.418+81_418+82delTT|1352|Ibtk|protein_coding|CODING|NM_001081282|3|1)</td></tr>
<tr><td>chr10</td><td>9812034</td><td>9812034</td><td>Stxbp5</td><td>T</td><td>TA</td><td></td><td>het</td><td>het</td><td>het</td><td>het</td><td>het</td><td></td><td>splice_region_variant</td><td>protein_coding</td><td>NM_001081344:c.1297_1298insT</td><td>splice_region_variant(LOW|||c.1297_1298insT|1116|Stxbp5|protein_coding|CODING|NM_001081344|12|1),intron_variant(MODIFIER|||c.1297-2_1297-3insT|1116|Stxbp5|protein_coding|CODING|NM_001081344|12|1)</td></tr>
<tr><td>chr10</td><td>14444202</td><td>14444202</td><td>Gpr126</td><td>A</td><td>ATT</td><td></td><td>het</td><td>het</td><td>het</td><td>het</td><td>het</td><td></td><td>intron_variant</td><td>protein_coding</td><td>NM_001002268:c.1344-54_1344-55insAA</td><td>intron_variant(MODIFIER|||c.1344-54_1344-55insAA|1165|Gpr126|protein_coding|CODING|NM_001002268|8|1)</td></tr>
<tr><td>chr10</td><td>14523042</td><td>14523042</td><td>Gpr126</td><td>C</td><td>CAAA</td><td></td><td>het</td><td>het</td><td>het</td><td>het</td><td>het</td><td></td><td>intron_variant</td><td>protein_coding</td><td>NM_001002268:c.103+12812_103+12811insTTT</td><td>intron_variant(MODIFIER|||c.103+12812_103+12811insTTT|1165|Gpr126|protein_coding|CODING|NM_001002268|2|1)</td></tr>
<tr><td>chr10</td><td>18470182</td><td>18470182</td><td>Nhsl1</td><td>CGTGT</td><td>C</td><td></td><td>het</td><td>het</td><td>het</td><td>het</td><td>het</td><td></td><td>intron_variant</td><td>protein_coding</td><td>NM_173390:c.203-2736_203-2733delGTGT</td><td>intron_variant(MODIFIER|||c.203-2736_203-2733delGTGT|1587|Nhsl1|protein_coding|CODING|NM_173390|1|1),intron_variant(MODIFIER|||c.58+18_58+21delGTGT|1583|Nhsl1|protein_coding|CODING|NM_001163592|1|1)</td></tr>
<tr><td>chr11</td><td>106830881</td><td>106830881</td><td>Smurf2</td><td>CTTTTTTT</td><td>C</td><td></td><td>het</td><td>het</td><td>hom</td><td>hom</td><td>hom</td><td></td><td>intron_variant</td><td>protein_coding</td><td>NM_025481:c.1748+32_1748+38delAAAAAAA</td><td>intron_variant(MODIFIER|||c.1748+32_1748+38delAAAAAAA|748|Smurf2|protein_coding|CODING|NM_025481|15|1)</td></tr>
<tr><td>chr12</td><td>29984749</td><td>29984749</td><td>Pxdn</td><td>T</td><td>TGGG</td><td>rs264717772</td><td>hom</td><td>hom</td><td>hom</td><td>hom</td><td>hom</td><td></td><td>intron_variant</td><td>protein_coding</td><td>NM_181395:c.722-117_722-116insGGG</td><td>intron_variant(MODIFIER|||c.722-117_722-116insGGG|1475|Pxdn|protein_coding|CODING|NM_181395|7|1)</td></tr>
<tr><td>chr12</td><td>50366266</td><td>50366266</td><td>Prkd1</td><td>G</td><td>GT</td><td></td><td>het</td><td>het</td><td>hom</td><td>hom</td><td>het</td><td></td><td>intron_variant</td><td>protein_coding</td><td>NM_008858:c.2085+36_2085+35insA</td><td>intron_variant(MODIFIER|||c.2085+36_2085+35insA|918|Prkd1|protein_coding|CODING|NM_008858|15|1)</td></tr>
<tr><td>chr12</td><td>50490135</td><td>50490135</td><td>Prkd1</td><td>CT</td><td>C</td><td></td><td>hom</td><td>hom</td><td>hom</td><td>het</td><td>het</td><td></td><td>intron_variant</td><td>protein_coding</td><td>NM_008858:c.259-95delA</td><td>intron_variant(MODIFIER|||c.259-95delA|918|Prkd1|protein_coding|CODING|NM_008858|1|1)</td></tr>
<tr><td>chr12</td><td>55410409</td><td>55410409</td><td>Psma6</td><td>GTT</td><td>G</td><td></td><td>hom</td><td>het</td><td>hom</td><td>hom</td><td>hom</td><td></td><td>intron_variant</td><td>protein_coding</td><td>NM_011968:c.409+128_409+129delTT</td><td>intron_variant(MODIFIER|||n.22+39694_22+39695delTT||Mir1198||NON_CODING|NR_035430|1|1),intron_variant(MODIFIER|||c.409+128_409+129delTT|246|Psma6|protein_coding|CODING|NM_011968|4|1)</td></tr>
<tr><td>chr12</td><td>56340178</td><td>56340178</td><td>Mbip</td><td>T</td><td>TA</td><td></td><td>hom</td><td>het</td><td>het</td><td>hom</td><td>hom</td><td></td><td>intron_variant</td><td>protein_coding</td><td>NM_145442:c.565+23_565+22insT</td><td>intron_variant(MODIFIER|||c.565+23_565+22insT|341|Mbip|protein_coding|CODING|NM_145442|4|1)</td></tr>
<tr><td>chr13</td><td>4078094</td><td>4078094</td><td>Akr1c14</td><td>GAGAGAGAG</td><td>G</td><td></td><td>het</td><td>hom</td><td>het</td><td>het</td><td>het</td><td></td><td>intron_variant</td><td>protein_coding</td><td>NM_134072:c.447+48_447+57delGAGAGAGAGA</td><td>intron_variant(MODIFIER|||c.447+48_447+57delGAGAGAGAGA|323|Akr1c14|protein_coding|CODING|NM_134072|4|1)</td></tr>
<tr><td>chr15</td><td>6837600</td><td>6837600</td><td>Osmr</td><td>GAAA</td><td>G</td><td></td><td>het</td><td>het</td><td>het</td><td>het</td><td>het</td><td></td><td>intron_variant</td><td>protein_coding</td><td>NM_011019:c.1122+89_1122+91delTTT</td><td>intron_variant(MODIFIER|||c.1122+89_1122+91delTTT|971|Osmr|protein_coding|CODING|NM_011019|8|1)</td></tr>
<tr><td>chr15</td><td>8121958</td><td>8121958</td><td>Nup155</td><td>TGTGTGTGT</td><td>C</td><td></td><td>het</td><td>het</td><td>het</td><td>het</td><td>hom</td><td></td><td>intron_variant</td><td>protein_coding</td><td>NM_133227:c.1094-97_1094-88delTGTGTGTGTG</td><td>intron_variant(MODIFIER|||c.1094-97_1094-88delTGTGTGTGTG|1391|Nup155|protein_coding|CODING|NM_133227|10|1)</td></tr>
<tr><td>chr15</td><td>10591698</td><td>10591698</td><td>Rai14</td><td>T</td><td>TACAC</td><td></td><td>het</td><td>het</td><td>het</td><td>het</td><td>hom</td><td></td><td>intron_variant</td><td>protein_coding</td><td>NM_001166408:c.737-83_737-84insGTGT</td><td>intron_variant(MODIFIER|||c.737-83_737-84insGTGT|979|Rai14|protein_coding|CODING|NM_001166408|9|1),intron_variant(MODIFIER|||c.737-83_737-84insGTGT|979|Rai14|protein_coding|CODING|NM_030690|9|1)</td></tr>
<tr><td>chr16</td><td>7223821</td><td>7223821</td><td>Rbfox1</td><td>T</td><td>TACAC</td><td></td><td>hom</td><td>het</td><td>hom</td><td>het</td><td>het</td><td></td><td>intron_variant</td><td>protein_coding</td><td>NM_183188:c.88-489_88-488insACAC</td><td>intron_variant(MODIFIER|||c.28-489_28-488insACAC|396|Rbfox1|protein_coding|CODING|NM_021477|5|1),intron_variant(MODIFIER|||c.88-489_88-488insACAC|417|Rbfox1|protein_coding|CODING|NM_183188|1|1)</td></tr>
<tr><td>chrX</td><td>49494731</td><td>49494731</td><td>Arhgap36</td><td>GCT</td><td>G</td><td></td><td>het</td><td>hom</td><td>het</td><td>het</td><td>het</td><td></td><td>intron_variant</td><td>protein_coding</td><td>NM_001081123:c.283+21_283+22delCT</td><td>intron_variant(MODIFIER|||c.283+21_283+22delCT|590|Arhgap36|protein_coding|CODING|NM_001081123|3|1)</td></tr>
</table>

Supplemental Table S4. List of variants consistent with a recessive inheritance pattern.

<table>
<tr><th rowspan="2">Chr</th><th rowspan="2">Start (bp)</th><th rowspan="2">End (bp)</th><th rowspan="2">Gene</th><th rowspan="2">Ref Allele</th><th rowspan="2">Alt allele</th><th rowspan="2">dbSNP</th><th colspan="6">Genotype</th><th rowspan="2">Annotation (primary transcript)</th><th rowspan="2">Annotation (alt transcript)</th><th rowspan="2">Transcript: Variant</th><th rowspan="2">Transcript isoforms with annotation</th></tr>
<tr><th>1153</th><th>68</th><th>942</th><th>1149</th><th>9655</th><th>1150</th></tr>
<tr><td>chr1</td><td>20814934</td><td>20814934</td><td>Mcm3</td><td>G</td><td>GGGA</td><td>rs242440541</td><td>hom</td><td>hom</td><td>hom</td><td>hom</td><td>hom</td><td>het</td><td>splice_region_variant</td><td>protein_coding</td><td>NM_008563:c.532_533insTCC</td><td>splice_region_variant(LOW|||c.532_533insTCC|812|Mcm3|protein_coding|CODING|NM_008563|4|1),intron_variant(MODIFIER|||c.532-7_532-8insTCC|812|Mcm3|protein_coding|CODING|NM_008563|4|1)</td></tr>
<tr><td>chr1</td><td>20990590</td><td>20990590</td><td>Efhc1</td><td>C</td><td>CTT</td><td>rs260911513</td><td>hom</td><td>hom</td><td>hom</td><td>hom</td><td>hom</td><td></td><td>intron_variant</td><td>protein_coding</td><td>NM_027974:c.1876-26_1876-25insTT</td><td>intron_variant(MODIFIER|||c.1876-26_1876-25insTT|648|Efhc1|protein_coding|CODING|NM_027974|10|1)</td></tr>
<tr><td>chr1</td><td>38812397</td><td>38812397</td><td>Lonrf2</td><td>AAC</td><td>A</td><td>rs226794629</td><td>hom</td><td>hom</td><td>hom</td><td>hom</td><td>hom</td><td></td><td>intron_variant</td><td>protein_coding</td><td>NM_001029878:c.653+145_653+146delGT</td><td>intron_variant(MODIFIER|||c.653+145_653+146delGT|518|Lonrf2|protein_coding|CODING|NM_001029878|5|1)</td></tr>
<tr><td>chr1</td><td>44175792</td><td>44175792</td><td>Ercc5</td><td>GTA</td><td>G</td><td>rs233333477</td><td>hom</td><td>hom</td><td>hom</td><td>hom</td><td>hom</td><td>het</td><td>intron_variant</td><td>protein_coding</td><td>NM_011729:c.2531-46_2531-45delTA</td><td>intron_variant(MODIFIER|||c.2531-46_2531-45delTA|1170|Ercc5|protein_coding|CODING|NM_011729|11|1)</td></tr>
<tr><td>chr1</td><td>45805341</td><td>45805341</td><td>Wdr75</td><td>G</td><td>GTT</td><td></td><td>hom</td><td>hom</td><td>hom</td><td>hom</td><td>hom</td><td></td><td>intron_variant</td><td>protein_coding</td><td>NM_028599:c.496-35_496-34insTT</td><td>intron_variant(MODIFIER|||c.496-35_496-34insTT|830|Wdr75|protein_coding|CODING|NM_028599|5|1)</td></tr>
<tr><td>chr1</td><td>59107615</td><td>59107615</td><td>Tmem237</td><td>C</td><td>CCACACA</td><td></td><td>hom</td><td>hom</td><td>hom</td><td>hom</td><td>hom</td><td>het</td><td>intron_variant</td><td>protein_coding</td><td>NM_001033449:c.894-88_894-89insTGTGTG</td><td>intron_variant(MODIFIER|||c.894-88_894-89insTGTGTG|413|Tmem237|protein_coding|CODING|NM_001033449|9|1),intron_variant(MODIFIER|||c.864-88_864-89insTGTGTG|403|Tmem237|protein_coding|CODING|NM_001037812|9|1)</td></tr>
<tr><td>chr1</td><td>64937781</td><td>64937781</td><td>Plekhm3</td><td>C</td><td>CCTGCTG</td><td></td><td>hom</td><td>hom</td><td>hom</td><td>hom</td><td>hom</td><td>het</td><td>inframe_insertion</td><td>protein_coding</td><td>NM_001039493:p.Gln176_Ala177insGlnGlnGln/c.528_529insCAGCAG</td><td>inframe_insertion(MODERATE||cag/cagCAGCAG|p.Gln176_Ala177insGlnGlnGln/c.528_529insCAGCAG|761|Plekhm3|protein_coding|CODING|NM_001039493|2|1)</td></tr>
<tr><td>chr1</td><td>75529223</td><td>75529223</td><td>Stk11ip</td><td>T</td><td>TGGAGGA</td><td></td><td>hom</td><td>hom</td><td>hom</td><td>hom</td><td>hom</td><td>het</td><td>inframe_insertion</td><td>protein_coding</td><td>NM_027886:p.Leu512_Glu513insLeuGluGlu/c.1536_1537insGGAGGA</td><td>inframe_insertion(MODERATE||ctg/ctGGAGGAg|p.Leu512_Glu513insLeuGluGlu/c.1536_1537insGGAGGA|1072|Stk11ip|protein_coding|CODING|NM_027886|14|1)</td></tr>
<tr><td>chr1</td><td>80578770</td><td>80578770</td><td>Dock10</td><td>TACACACAC</td><td>T</td><td></td><td>hom</td><td>hom</td><td>hom</td><td>hom</td><td>hom</td><td></td><td>intron_variant</td><td>protein_coding</td><td>NM_175291:c.1686-70_1686-63delGTGTGTGT</td><td>intron_variant(MODIFIER|||c.1686-70_1686-63delGTGTGTGT|2187|Dock10|protein_coding|CODING|NM_175291|14|1)</td></tr>
<tr><td>chr1</td><td>88056440</td><td>88056440</td><td>Ugt1a10</td><td>G</td><td>GTTTTT</td><td></td><td>hom</td><td>hom</td><td>hom</td><td>hom</td><td>hom</td><td></td><td>intron_variant</td><td>protein_coding</td><td>NM_201641:c.855+105_855+106insTTTTT</td><td>intron_variant(MODIFIER|||c.855+105_855+106insTTTTT|530|Ugt1a10|protein_coding|CODING|NM_201641|1|1)</td></tr>
<tr><td>chr1</td><td>135371901</td><td>135371901</td><td>Shisa4</td><td>A</td><td>AAAAAC</td><td>rs246361051</td><td>hom</td><td>hom</td><td>hom</td><td>hom</td><td>hom</td><td></td><td>3_prime_UTR_variant</td><td>protein_coding</td><td>NM_175259:c.*48_*49insGTTTT</td><td>3_prime_UTR_variant(MODIFIER||48|c.*48_*49insGTTTT|197|Shisa4|protein_coding|CODING|NM_175259|5|1)</td></tr>
<tr><td>chr1</td><td>151368380</td><td>151368380</td><td>Swt1</td><td>T</td><td>TTTTTTTTTC</td><td>rs216922920</td><td>hom</td><td>hom</td><td>hom</td><td>hom</td><td>hom</td><td></td><td>3_prime_UTR_variant</td><td>protein_coding</td><td>NM_025819:c.*78_*79insGAAAAAAAA</td><td>3_prime_UTR_variant(MODIFIER||78|c.*78_*79insGAAAAAAAA|907|Swt1|protein_coding|CODING|NM_025819|20|1)</td></tr>
<tr><td>chr1</td><td>151419965</td><td>151419965</td><td>Swt1</td><td>TG</td><td>T</td><td></td><td>hom</td><td>hom</td><td>hom</td><td>hom</td><td>hom</td><td>het</td><td>intron_variant</td><td>protein_coding</td><td>NM_025819:c.225+134delC</td><td>intron_variant(MODIFIER|||c.225+134delC|907|Swt1|protein_coding|CODING|NM_025819|4|1)</td></tr>
<tr><td>chr1</td><td>154421318</td><td>154421318</td><td>Cacna1e</td><td>CT</td><td>C</td><td>rs387429597</td><td>hom</td><td>hom</td><td>hom</td><td>hom</td><td>hom</td><td>het</td><td>intron_variant</td><td>protein_coding</td><td>NM_009782:c.5149-164delA</td><td>intron_variant(MODIFIER|||c.5149-164delA|2273|Cacna1e|protein_coding|CODING|NM_009782|37|1)</td></tr>
<tr><td>chr1</td><td>172133959</td><td>172133959</td><td>Pex19</td><td>C</td><td>CTTT</td><td></td><td>hom</td><td>hom</td><td>hom</td><td>hom</td><td>hom</td><td></td><td>intron_variant</td><td>protein_coding</td><td>NM_023041:c.772-40_772-39insTTT</td><td>intron_variant(MODIFIER|||c.496-40_496-39insTTT|207|Pex19|protein_coding|CODING|NM_001159525|4|1),intron_variant(MODIFIER|||c.772-40_772-39insTTT|299|Pex19|protein_coding|CODING|NM_023041|6|1)</td></tr>
<tr><td>chr1</td><td>184795309</td><td>184795309</td><td>Marc1</td><td>G</td><td>GAC</td><td>rs239455198</td><td>hom</td><td>hom</td><td>hom</td><td>hom</td><td>hom</td><td>het</td><td>splice_region_variant</td><td>protein_coding</td><td>NM_001081361:c.831_832insGT</td><td>splice_region_variant(LOW|||c.831_832insGT|342|Marc1|protein_coding|CODING|NM_001081361|7|1),intron_variant(MODIFIER|||c.831-4_831-5insGT|342|Marc1|protein_coding|CODING|NM_001081361|7|1)</td></tr>
<tr><td>chr4</td><td>43018347</td><td>43018347</td><td>Pigo</td><td>GA</td><td>G</td><td>rs214864308</td><td>hom</td><td>hom</td><td>hom</td><td>hom</td><td>hom</td><td>het</td><td>intron_variant</td><td>protein_coding</td><td>NM_020035:c.3106-130delT</td><td>intron_variant(MODIFIER|||c.3106-130delT|1101|Pigo|protein_coding|CODING|NM_020035|8|1)</td></tr>
<tr><td>chr5</td><td>137302316</td><td>137302316</td><td>Srrt</td><td>T</td><td>TG</td><td>rs219583007</td><td>hom</td><td>hom</td><td>hom</td><td>hom</td><td>hom</td><td>het</td><td>splice_region_variant</td><td>protein_coding</td><td>NM_031405:c.399_400insC</td><td>splice_region_variant(LOW|||c.399_400insC|868|Srrt|protein_coding|CODING|NM_001109909|4|1),splice_region_variant(LOW|||c.399_400insC|864|Srrt|protein_coding|CODING|NM_001109910|4|1),splice_region_variant(LOW|||c.399_400insC|875|Srrt|protein_coding|CODING|NM_031405|4|1),intron_variant(MODIFIER|||c.399-2_399-3insC|868|Srrt|protein_coding|CODING|NM_001109909|4|1),intron_variant(MODIFIER|||c.399-2_399-3insC|864|Srrt|protein_coding|CODING|NM_001109910|4|1),intron_variant(MODIFIER|||c.399-2_399-3insC|875|Srrt|protein_coding|CODING|NM_031405|4|1)</td></tr>
<tr><td>chr5</td><td>137428572</td><td>137428572</td><td>Zan</td><td>AC</td><td>A</td><td>rs387507091</td><td>hom</td><td>hom</td><td>hom</td><td>hom</td><td>hom</td><td>het</td><td>intron_variant</td><td>protein_coding</td><td>NM_011741:c.7360-50delG</td><td>intron_variant(MODIFIER|||c.7360-50delG|5420|Zan|protein_coding|CODING|NM_011741|36|1)</td></tr>
<tr><td>chr5</td><td>139753357</td><td>139753357</td><td>Ints1</td><td>T</td><td>TAC</td><td>rs243186654</td><td>hom</td><td>hom</td><td>hom</td><td>hom</td><td>hom</td><td>het</td><td>intron_variant</td><td>protein_coding</td><td>NM_026748:c.6166+104_6166+103insGT</td><td>intron_variant(MODIFIER|||c.6166+104_6166+103insGT|2222|Ints1|protein_coding|CODING|NM_026748|42|1)</td></tr>
<tr><td>chr5</td><td>143721062</td><td>143721062</td><td>Usp42</td><td>TAA</td><td>T</td><td>rs249277515</td><td>hom</td><td>hom</td><td>hom</td><td>hom</td><td>hom</td><td></td><td>intron_variant</td><td>protein_coding</td><td>NM_029749:c.875+84_875+85delTT</td><td>intron_variant(MODIFIER|||c.875+84_875+85delTT|1324|Usp42|protein_coding|CODING|NM_029749|8|1)</td></tr>
<tr><td>chr5</td><td>147682149</td><td>147682149</td><td>Flt1</td><td>CT</td><td>C</td><td></td><td>hom</td><td>hom</td><td>hom</td><td>hom</td><td>hom</td><td>het</td><td>intron_variant</td><td>protein_coding</td><td>NM_010228:c.517-143delA</td><td>intron_variant(MODIFIER|||c.517-143delA|1333|Flt1|protein_coding|CODING|NM_010228|4|1)</td></tr>
<tr><td>chr5</td><td>150260368</td><td>150260368</td><td>Fry</td><td>A</td><td>AATT</td><td></td><td>hom</td><td>hom</td><td>hom</td><td>hom</td><td>hom</td><td>het</td><td>intron_variant</td><td>protein_coding</td><td>NM_172887:c.70+31_70+32insATT</td><td>intron_variant(MODIFIER|||c.70+31_70+32insATT|3020|Fry|protein_coding|CODING|NM_172887|1|1)</td></tr>
<tr><td>chr6</td><td>93701094</td><td>93701094</td><td>Magi1</td><td>AAAC</td><td>A</td><td></td><td>hom</td><td>hom</td><td>hom</td><td>hom</td><td>hom</td><td></td><td>intron_variant</td><td>protein_coding</td><td>NM_001029850:c.2568+193_2568+195delGTT</td><td>intron_variant(MODIFIER|||c.2568+193_2568+195delGTT|1471|Magi1|protein_coding|CODING|NM_001029850|16|1),intron_variant(MODIFIER|||c.1884+193_1884+195delGTT|1255|Magi1|protein_coding|CODING|NM_001083320|15|1),intron_variant(MODIFIER|||c.1887+193_1887+195delGTT|1020|Magi1|protein_coding|CODING|NM_001083321|15|1),intron_variant(MODIFIER|||c.2445+193_2445+195delGTT|1171|Magi1|protein_coding|CODING|NM_010367|14|1)</td></tr>
<tr><td>chr6</td><td>119206643</td><td>119206643</td><td>Dcp1b</td><td>CTT</td><td>C</td><td></td><td>hom</td><td>hom</td><td>hom</td><td>hom</td><td>hom</td><td>het</td><td>intron_variant</td><td>protein_coding</td><td>NM_001033379:c.645+46_645+47delTT</td><td>intron_variant(MODIFIER|||c.645+46_645+47delTT|578|Dcp1b|protein_coding|CODING|NM_001033379|6|1)</td></tr>
<tr><td>chr6</td><td>120428021</td><td>120428021</td><td>Kdm5a</td><td>T</td><td>TA</td><td></td><td>hom</td><td>hom</td><td>hom</td><td>hom</td><td>hom</td><td>het</td><td>intron_variant</td><td>protein_coding</td><td>NM_145997:c.4074+150_4074+151insA</td><td>intron_variant(MODIFIER|||c.4074+150_4074+151insA|1690|Kdm5a|protein_coding|CODING|NM_145997|24|1)</td></tr>
<tr><td>chr6</td><td>125140883</td><td>125140883</td><td>Nop2</td><td>AGGGCCT</td><td>A</td><td>rs239980921</td><td>hom</td><td>hom</td><td>hom</td><td>hom</td><td>hom</td><td>het</td><td>splice_region_variant</td><td>protein_coding</td><td>NM_138747:c.1393_1398delGGGCCT</td><td>splice_region_variant(LOW|||c.1393_1398delGGGCCT|794|Nop2|protein_coding|CODING|NM_138747|13|1),intron_variant(MODIFIER|||c.1392+5_1392+10delGGGCCT|794|Nop2|protein_coding|CODING|NM_138747|13|1)</td></tr>
<tr><td>chr11</td><td>74993946</td><td>74993946</td><td>Smg6</td><td>T</td><td>TA</td><td>rs212679580</td><td>hom</td><td>hom</td><td>hom</td><td>hom</td><td>hom</td><td>het</td><td>intron_variant</td><td>protein_coding</td><td>NM_001002764:c.2866+3732_2866+3733insA</td><td>intron_variant(MODIFIER|||c.2866+3732_2866+3733insA|1418|Smg6|protein_coding|CODING|NM_001002764|10|1)</td></tr>
<tr><td>chr12</td><td>29984749</td><td>29984749</td><td>Pxdn</td><td>T</td><td>TGGG</td><td>rs264717772</td><td>hom</td><td>hom</td><td>hom</td><td>hom</td><td>hom</td><td></td><td>intron_variant</td><td>protein_coding</td><td>NM_181395:c.722-117_722-116insGGG</td><td>intron_variant(MODIFIER|||c.722-117_722-116insGGG|1475|Pxdn|protein_coding|CODING|NM_181395|7|1)</td></tr>
<tr><td>chr17</td><td>3414780</td><td>3414780</td><td>Tiam2</td><td>G</td><td>C</td><td>rs33171387</td><td>hom</td><td>hom</td><td>hom</td><td>hom</td><td>hom</td><td>het</td><td>missense_variant</td><td>MISSENSE</td><td>NM_001122998:p.Glu261Asp/c.783G>C</td><td>missense_variant(MODERATE|MISSENSE|gaG/gaC|p.Glu261Asp/c.783G>C|1715|Tiam2|protein_coding|CODING|NM_001122998|3|1),missense_variant(MODERATE|MISSENSE|gaG/gaC|p.Glu261Asp/c.783G>C|1715|Tiam2|protein_coding|CODING|NM_011878|3|1)</td></tr>
</table>

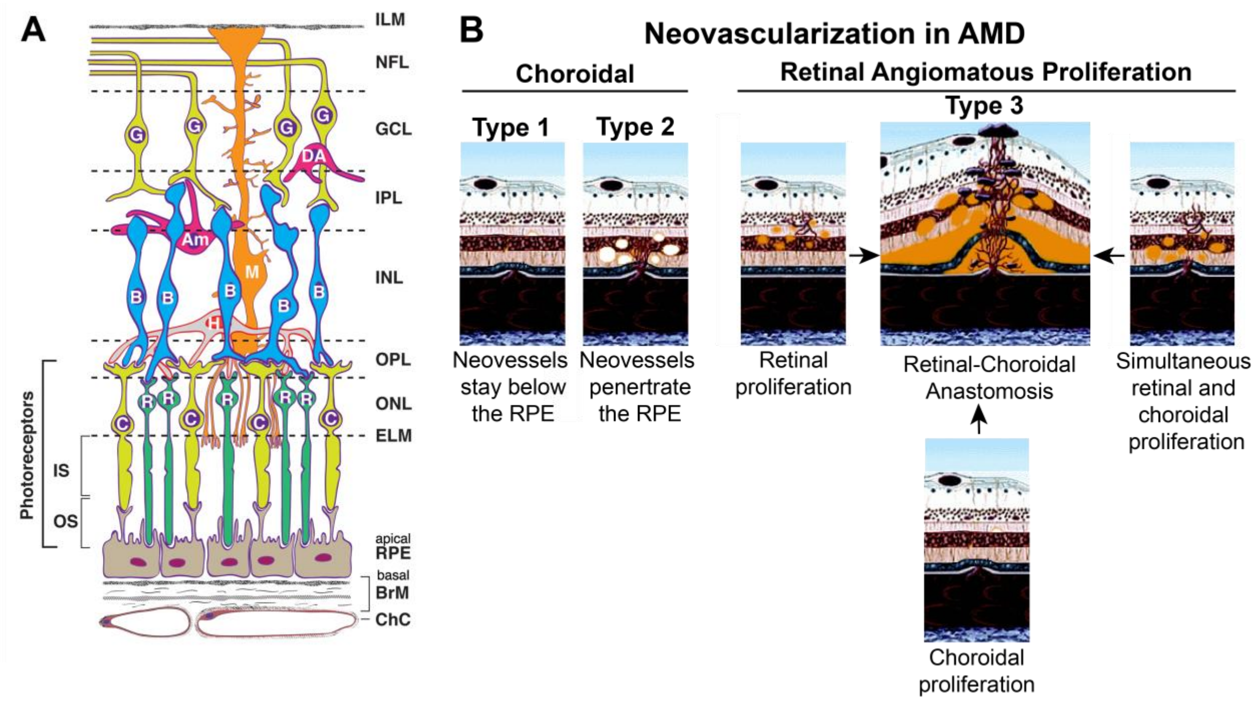

Figure S1. The retina and age-related macular degeneration. A, chorioretinal cells and layers. Taken from Zheng *et al*. 2012, PLos One, and modified. Cells (from bottom to top): RPE, retinal pigment epithelium (nurse cells to the photoreceptors); C, cone photoreceptor; R, rod photoreceptor; H, horizontal cell (interneuron); B, bipolar cell (interneuron); M, Müller cell (radial glial cell); Am, amacrine cell (interneuron); DA, displaced amacrine cell (interneuron); G, ganglion cell (output neuron). Müller cells (M) extend almost the width of the retina; their apical processes form the ELM, and their foot processes partially form the ILM. Layers (from bottom to top): ChC, choriocapillaris (capillary bed for the RPE and photoreceptors); BrM, Bruch's membrane (vessel wall and RPE substratum); ELM, external limiting membrane (junctional complexes); ONL, outer nuclear layer; OPL, outer plexiform layer (synapses); INL, inner nuclear layer; IPL, inner plexiform layer; GCL, ganglion cell layer; NFL, nerve fiber layer (ganglion cell axons); ILM, inner limiting membrane. Non-photoreceptor layers of the retina are supplied by the retinal circulation (not shown). Graphics by D. Fisher; inspired by Figure 4-2 of Ryan SJ, editor. **B**, Schematic representation of neovascularization in age-related macular degeneration (AMD). Taken with permission from Yannuzzi et al. 2008, Retina, and modified. See main text for details.

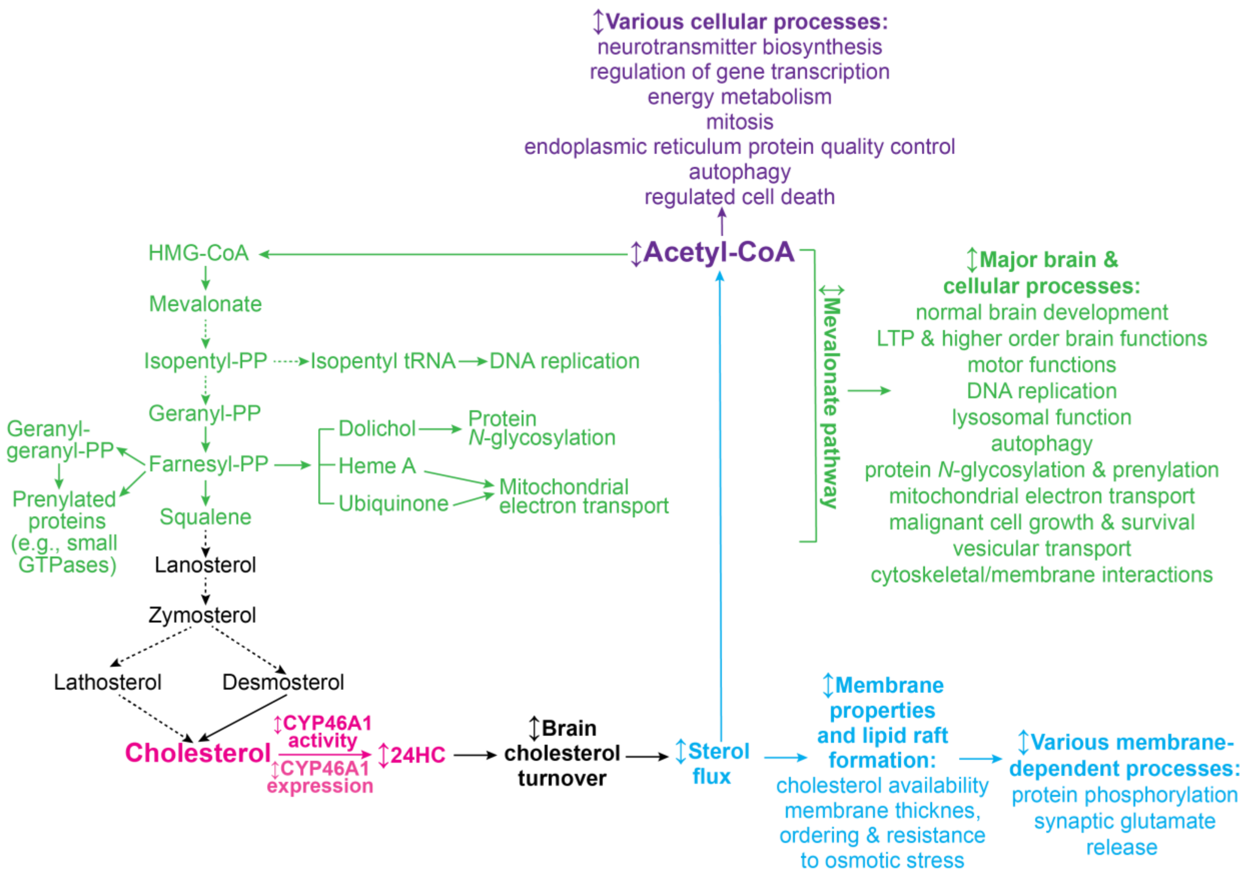

Figure S2. Schematic representation of the three primary processes (the unifying mechanisms) that can integrate a variety of CYP46A1 targeting effects. These are mevalonate pathway (green), sterol flux (blue), and acetyl-CoA production (violet). See main text for details. The initial event, CYP46A1-mediated cholesterol 24-hydroxylation, is colored in magenta. Dashed arrows indicate multiple steps; ↕: the up-down arrow indicates modulation (increase or decrease); HMG: 3-hydroxy-3-methylglutaryl; PP, pyrophosphate. Taken from Pikuleva, 2021, *Explor Neuroprot Ther*.
